# Physician Emigration from Sub-Saharan Africa to the United States: Analysis of the 2011 AMA Physician Masterfile

**DOI:** 10.1371/journal.pmed.1001513

**Published:** 2013-09-17

**Authors:** Akhenaten Benjamin Siankam Tankwanchi, Çağlar Özden, Sten H. Vermund

**Affiliations:** 1 Department of Human and Organizational Development, Peabody School of Education, Vanderbilt University, Nashville, Tennessee, United States of America; 2 Development Research Group, The World Bank, Washington (D.C.), United States of America; 3 Vanderbilt Institute for Global Health, Vanderbilt University, Nashville, Tennessee, United States of America; 4 Department of Pediatrics, School of Medicine, Vanderbilt University, Nashville, Tennessee, United States of America; Columbia University Mailman School of Public Health, United States of America

## Abstract

**Background:**

The large-scale emigration of physicians from sub-Saharan Africa (SSA) to high-income nations is a serious development concern. Our objective was to determine current emigration trends of SSA physicians found in the physician workforce of the United States.

**Methods and Findings:**

We analyzed physician data from the World Health Organization (WHO) Global Health Workforce Statistics along with graduation and residency data from the 2011 American Medical Association Physician Masterfile (AMA-PM) on physicians trained or born in SSA countries who currently practice in the US. We estimated emigration proportions, year of US entry, years of practice before emigration, and length of time in the US. According to the 2011 AMA-PM, 10,819 physicians were born or trained in 28 SSA countries. Sixty-eight percent (*n* = 7,370) were SSA-trained, 20% (*n* = 2,126) were US-trained, and 12% (*n* = 1,323) were trained outside both SSA and the US. We estimated active physicians (age ≤70 years) to represent 96% (*n* = 10,377) of the total. Migration trends among SSA-trained physicians increased from 2002 to 2011 for all but one principal source country; the exception was South Africa whose physician migration to the US decreased by 8% (−156). The increase in last-decade migration was >50% in Nigeria (+1,113) and Ghana (+243), >100% in Ethiopia (+274), and >200% (+244) in Sudan. Liberia was the most affected by migration to the US with 77% (*n* = 175) of its estimated physicians in the 2011 AMA-PM. On average, SSA-trained physicians have been in the US for 18 years. They practiced for 6.5 years before US entry, and nearly half emigrated during the implementation years (1984–1999) of the structural adjustment programs.

**Conclusion:**

Physician emigration from SSA to the US is increasing for most SSA source countries. Unless far-reaching policies are implemented by the US and SSA countries, the current emigration trends will persist, and the US will remain a leading destination for SSA physicians emigrating from the continent of greatest need.

*Please see later in the article for the Editors' Summary*

## Abstract 2

Siankam Tankwanchi and colleagues used the AMA Physician Masterfile and the WHO Global Health Workforce Statistics on physicians in sub-Saharan Africa to determine trends in physician emigration to the United States.

*Please see later in the article for the Editors' Summary*

## Editors' Summary

**Background:**

Population growth and aging and increasingly complex health care interventions, as well as existing policies and market forces, mean that many countries are facing a shortage of health care professionals. High-income countries are addressing this problem in part by encouraging the immigration of foreign health care professionals from low- and middle-income countries. In the US, for example, international medical graduates (IMGs) can secure visas and permanent residency by passing examinations provided by the Educational Commission of Foreign Medical Graduates and by agreeing to provide care in areas that are underserved by US physicians. Inevitably, the emigration of physicians from low- and middle-income countries undermines health service delivery in the emigrating physicians' country of origin because physician supply is already inadequate in those countries. Physician emigration from sub-Saharan Africa, which has only 2% of the global physician workforce but a quarter of the global burden of disease, is particularly worrying. Since 1970, as a result of large-scale emigration and limited medical education, there has been negligible or negative growth in the density of physicians in many countries in sub-Saharan Africa. In Liberia, for example, in 1973, there were 7.76 physicians per 100,000 people but by 2008 there were only 1.37 physicians per 100,000 people; in the US, there are 250 physicians per 100,000 people.

**Why Was This Study Done?:**

Before policy proposals can be formulated to address global inequities in physician distribution, a clear picture of the patterns of physician emigration from resource-limited countries is needed. In this study, the researchers use data from the 2011 American Medical Association Physician Masterfile (AMA-PM) to investigate the “brain drain” of physicians from sub-Saharan Africa to the US. The AMA-PM collects annual demographic, academic, and professional data on all residents (physicians undergoing training in a medical specialty) and licensed physicians who practice in the US.

**What Did the Researchers Do and Find?:**

The researchers used data from the World Health Organization (WHO) Global Health Workforce Statistics and graduation and residency data from the 2011 AMA-PM to estimate physician emigration rates from sub-Saharan African countries, year of US entry, years of service provided before emigration to the US, and length of time in the US. There were 10,819 physicians who were born or trained in 28 sub-Saharan African countries in the 2011 AMA-PM. By using a published analysis of the 2002 AMA-PM, the researchers estimated that US immigration among sub-Saharan African-trained physicians had increased over the past decade for all the countries examined except South Africa, where physician emigration had decreased by 8%. Overall, the number of sub-Saharan African IMGs in the US had increased by 38% since 2002. More than half of this increase was accounted for by Nigerian IMGs. Liberia was the country most affected by migration of its physicians to the US—77% of its estimated 226 physicians were in the 2011 AMA-PM. On average, sub-Saharan African IMGs had been in the US for 18 years and had practiced for 6.5 years before emigration. Finally, nearly half of the sub-Saharan African IMGs had migrated to US between 1984 and 1995, years during which structural adjustment programs, which resulted in deep cuts to public health care services, were implemented in developing countries by international financial institutions as conditions for refinancing.

**What Do These Findings Mean?:**

Although the sub-Saharan African IMGs in the 2011 AMA-PM only represent about 1% of all the physicians and less than 5% of the IMGs in the AMA-PM, these findings reveal a major loss of physicians from sub-Saharan Africa. They also suggest that emigration of physicians from sub-Saharan Africa is a growing problem and is likely to continue unless job satisfaction for physicians is improved in their country of origin. Moreover, because the AMA-PM only lists physicians who qualify for a US residency position, more physicians may have moved from sub-Saharan Africa to the US than reported here and may be working in other jobs incommensurate with their medical degrees (“brain waste”). The researchers suggest that physician emigration from sub-Saharan Africa to the US reflects the complexities in the labor markets for health care professionals in both Africa and the US and can be seen as low- and middle-income nations subsidizing the education of physicians in high-income countries. Policy proposals to address global inequities in physician distribution will therefore need both to encourage the recruitment, training, and retention of health care professionals in resource-limited countries and to persuade high-income countries to train more home-grown physicians to meet the needs of their own populations.

**Additional Information:**

Please access these websites via the online version of this summary at http://dx.doi.org/10.1371/journal.pmed.1001513.

- The Foundation for Advancement of International Medical Education and Research is a non-profit foundation committed to improving world health through education that was established in 2000 by the Educational Commission for Foreign Medical Graduates
- The Global Health Workforce Alliance is a partnership of national governments, civil society, international agencies, finance institutions, researchers, educators, and professional associations dedicated to identifying, implementing and advocating for solutions to the chronic global shortage of health care professionals (available in several languages)
- Information on the American Medical Association Physician Masterfile and the providers of physician data lists is available via the American Medical Associations website
- The World Health Organization (WHO) annual World Health Statistics reports present the most recent health statistics for the WHO Member States
- The Medical Education Partnership Initiative is a US-sponsored initiative that supports medical education and research in sub-Saharan African institutions, aiming to increase the quantity, quality, and retention of graduates with specific skills addressing the health needs of their national populations
- Capacity*Plus* is the USAID-funded global project uniquely focused on the health workforce needed to achieve the Millennium Development Goals
- Seed Global Health cultivates the next generation of health professionals by allying medical and nursing volunteers with their peers in resource-limited settings
- "America is Stealing the Worlds Doctors", a 2012 *New York Times* article by Matt McAllester, describes the personal experience of a young doctor who emigrated from Zambia to the US
- Path to United States Practice Is Long Slog to Foreign Doctors, a 2013 New York Times article by Catherine Rampell, describes the hurdles that immigrant physicians face in practicing in the US

## Introduction

The large-scale emigration of physicians from sub-Saharan Africa (SSA) to high income countries undermines the quality and delivery of health services in the resource-constrained origin countries. The World Health Organization (WHO) has emphasized the importance of an adequate number of qualified health care personnel for the effective delivery of health services [1]–[3]. As the late former WHO Director-General LEE Jong-Wook noted, “it takes a considerable investment of time and money to train health workers,” and when the latter emigrate, “there is a loss of hope and a loss of years of investment” [4].

Since 1970, the growth in physician density has been negligible or even negative in a significant number of SSA countries (Table 1) [5]–[11]. Liberia is one of the most dramatic examples. With only 51 physicians in 2008 for a largely rural population of almost 4 million people [5], Liberia has had one of the lowest physician-to-patient ratios in the world (1.37 physician per 100,000 people). Yet, 35 y earlier in 1973, there were 132 physicians in Liberia when the population was 1.7 million people [6], a 6-fold proportionate loss of coverage. Since its independence over 160 y ago, there has been only one medical school in Liberia [7]–[9]. Moreover, the long and ruinous civil wars that wreaked havoc in Liberia in the late 1980s and early 2000 destroyed scarce human resources and physical infrastructures, triggering a major brain drain from Africa's oldest republic [12].

**Table 1:** Changes in physician-to-population ratios (density) and medical schools in selected African and non-African countries, ranked by change in physician density.

Country	Circa 1970^a^				Circa 2010^a^				Change over Time		
	Population (in 1,000)	Physicians (*n*)	Density (100K people)	Medical Schools (*n*)	Population (in 1,000)	Physicians (*n*)	Density (100K people)	Medical Schools (*n*)	Physician Density (absolute)	Physician Density (%)	Medical Schools (*n*)
Liberia	1,397	132	9.4	1	4,190	51	1.2	1	−8.2	−87.1%	0
Tanzania	14,354	576	4.0	1	47,783	300	0.6	5	−3.4	−84.4%	4
Zimbabwe	5,515	1,035	18.8	1	13,724	827	6.0	1	−12.7	−67.9%	0
Mozambique	9,304	510	5.5	1	25,203	548	2.2	4	−3.3	−60.3%	3
Sierra Leone	2,789	149	5.3	0	5,979	136	2.3	1	−3.1	−57.4%	1
Zambia	4,248	527	12.4	1	14,075	836	5.9	1	−6.5	−52.1%	0
Somalia	3,667	193	5.3	1	10,195	300	2.9	2	−2.3	−44.1%	1
Djibouti	180	52	28.9	0	906	185	20.4	1	−8.5	−29.3%	1
Congo	1,272	163	12.8	1	4,337	401	9.2	1	−3.6	−27.8%	0
Niger	4,841	109	2.3	1	17,157	288	1.7	1	−0.6	−25.4%	0
Ghana	8,789	856	9.7	2	25,366	2,033	8.0	4	−1.7	−17.7%	2
Senegal	4,318	281	6.5	1	13,726	741	5.4	4	−1.1	−17.04%	3
Guinea-Bissau	620	55	8.9	0	1,664	124	7.5	1	−1.4	−16.0%	1
Lesotho	1,067	50	4.7	0	2,052	89	4.3	0	−0.3	−7.4%	0
Togo	1,964	95	4.8	1	6,643	349	5.3	1	0.4	8.6%	0
Swaziland	455	54	11.9	0	1,231	173	14.1	0	2.2	18.4%	0
South Africa	22,740	12,060	53.0	7	52,386	38,236	73.0	8	20.0	37.6%	1
Ethiopia	29,469	374	1.3	2	91,729	2,152	2.3	12	1.1	84.9%	10
Angola	5,606	383	6.8	1	20,821	2,946	14.1	7	7.3	107.1%	6
Rwanda	3,769	77	2.0	1	11,458	568	5.0	1	2.9	142.6%	0
Gambia	485	19	3.9	1	1,791	175	9.8	1	5.9	149.4%	0
Nigeria	59,607	2,343	3.9	12	169,000	55,376	32.8	25	28.8	733.6%	13
**Sub-Saharan Africa**	**287,856**	**25,504**	**8.9**	**53**	**913,302**	**150,305**	**16.5**	**109**	**7.6**	**85.7%**	**56**
Canada	22,479	37,277	165.8	16	34,838	69,699	200.1	17	34.2	20.6%	1
Brazil	58,854	59,573	101.2	77	199,000	341,849	171.8	90	70.6	69.7%	13
USA	211,909	338,111	159.6	126	318,000	909,749	286.1	147	126.5	79.3%	21
UK	55,968	75,141	134.3	26	62,783	172,553	274.8	33	140.6	104.7%	7
Belgium	9,757	16,476	168.9	7	11,090	39,690	357.9	11	189.0	111.9%	4
Australia	12,959	17,972	138.7	8	23,050	81,639	354.2	21	215.5	155.4%	13
South Korea	32,905	16,377	49.8	19	49,003	98,293	200.6	52	150.8	303.0%	33
Cuba	8,074	7,000	86.7	7	11,271	76,506	678.8	14	592.1	682.9%	7

Data sources: World Health Organization [5]; World Health Organization [6]; Mullan et al. [7]; Foundation for Advancement of International Medical Education and Research [8]; University of Copenhagen and World Health Organization [9]; Redi-Med Data [10]; United States Census Bureau [11].

a Circa 1970, 1969–1976; circa 2010, 2003–2012.

However, civil war does not explain the negative growth of physician density in relatively peaceful, politically stable, and steadily growing countries like Tanzania or Zambia, both of which were listed in 2011 among the world's ten fastest-growing economies by *The Economist* [13]. Yet, they have experienced, respectively, a 59% and 83% proportionate loss of physician coverage between the early 1970s and mid-2010s (Table 1). The extremely low physician densities in most SSA countries are exacerbated by limited medical education capacity [7],[14],[15], as well as large-scale emigration of physicians [16]–[28] in the midst of widespread communicable disease transmission and the rapid growth of non-communicable diseases [29]–[31]. Data from Clemens and Pettersson [16] suggest that Tanzania and Zambia, respectively, had 52% and 57% of their physicians living abroad between 1995 and 2001. The authors reported 1,264 physicians to be living in Tanzania in 1995 [16]. In their 2004 publication [19], Hagopian et al. reported 1,384 physicians in Tanzania for a population of nearly 38 million. If the latest WHO estimate of the Tanzanian active physician workforce (*n* = 300 physicians in 2006) is credible, this implies that Tanzania's health system has lost 1,084 (78.3%) active physicians in <10 y. Thus, Tanzania's physician density has effectively decreased from 4.1 physicians per 100,000 population to 0.69 physician per 100,000 population, amounting to a 5-fold loss of physician coverage in Tanzania between 1995 and 2004.

While the international migration choices of health care workers from SSA are becoming increasingly diverse, the two most prominent destinations are the United Kingdom and the United States [16]–[28]. Using the 2002 American Medical Association Physician Masterfile (AMA-PM), Hagopian et al. identified 5,334 US-based international medical graduates (IMGs) who received their medical degrees from SSA-based medical schools (hereafter SSA-IMGs). The leading countries were Nigeria, South Africa, Ghana, and Ethiopia. They further recorded 2,151 SSA-IMGs in Canada and estimated the combined figure of SSA-IMGs practicing in North America to represent over 9% of the stock of physicians available in SSA at the time [19]. Similarly, Mullan computed “The Metrics of the Physician Brain Drain” [20] by using the 2004 AMA-PM, identifying an aggregate total of 13,272 SSA-IMGs in the AMA-PM (US), the National Health Service (UK), the Southam Medical Database of the Canadian Institute for Health Information, the Canadian Post-MD Education Registry of the Association of Faculties of Medicine, and the Australian Institute of Health and Welfare. In this analysis, the SSA region had the highest rate of emigration, though absolute numbers of physician émigrés are higher from Asia and Latin America where there are much higher physician populations (notably from India, Pakistan, Philippines, and the Caribbean).

It is necessary to account for all SSA émigré physicians, including those educated outside SSA, to get an accurate picture of their migration profiles. The studies by Hagopian et al. [19] and Mullan [20] did not include the substantial number of African-born physicians trained internationally but now practicing in the US. This omission, typical also in other analyses [21],[22], does not permit an assessment of how many African physicians trained overseas are now unavailable to their home countries. In contrast, Western-educated physicians who practice, teach, and conduct research in medical schools across Africa are an integral part of the national physician counts reported by WHO [5], and are included in denominator data for physician emigration rates.

Clemens and Pettersson attempted to address the above limitation in their “New Data on African Health Professionals Abroad” [16]. Contrary to previous studies, which used location of medical education as a proxy for country of origin, Clemens and Pettersson identified the country of origin of medical expatriates by their place of birth. They observed that almost 65,000 African-born émigré physicians and nearly 70,000 African-born émigré professional nurses were practicing in eight Organization of Economic Cooperation and Development (OECD) high-income countries and in South Africa. Interestingly, while South Africa has been losing its own physicians and nurses to wealthier OECD countries, South Africa has also served as the main destination for African physicians who migrated regionally, that is, from one SSA country to another. Excluding SSA physicians and nurses who immigrated to South Africa, there were 35,000 SSA-born physicians and 53,000 SSA-born professional nurses identified by Clemens and Pettersson in eight OECD countries, representing one-fifth of SSA-born physicians and one-tenth of SSA-born professional nurses. SSA harbors about 14% of the world's population, but has only 3% of the world's health professionals [2], of whom 17.5% had emigrated by 2005. Clemens and Pettersson's findings indicated that the UK was the destination for the largest proportion of SSA medical emigrants, representing 10% (*n* = 13,350) of SSA-born physicians and 4% (*n* = 20,372) of SSA-born nurses. They further identified 6.4% (*n* = 8,558) physicians and 4% (*n* = 19,545) nurses from the SSA region in the US [16].

Clemens and Pettersson captured larger numbers of SSA émigré health professionals than reported in previous studies using the AMA-PM data. However, their analysis conflated émigré physicians trained by origin African countries and those trained internationally. They provided no information on the percentage comprising each group. Also, the use of the 2000 US Census data suggests that almost 9 y had elapsed from the time of data collection to the time the authors published their study. Since US census data are self-reported, IMGs who did not gain admission into US residency may or may not identify as medical doctors when completing US census surveys. Indeed, a cross-section of IMGs hoping to obtain a license to practice in the US do not pass the Educational Commission for Foreign Medical Graduates (ECFMG) certification, a requirement for IMGs' admission into US graduate medical education, the pathway through which one becomes licensed to practice medicine in the US [32]. A number of SSA-trained physicians currently living in the US are probably among these unsuccessful applicants. However, no known study has estimated their number nor examined their migration profile or encore/alternative careers in the US.

The US consistently reports significantly more African physicians than the UK when the “country of medical education” is used to define SSA physicians. For example, Mullan [20] counted 2,392 Nigerian-trained physicians in the US compared to 1,529 in the UK. Likewise, Hagopian et al. [19] reported 5,334 SSA-trained physicians in the US as opposed to 3,451 SSA-trained physicians in the UK. But when “country of birth” is used as the selection criterion, the UK has higher numbers as evidenced by Clemens and Pettersson [16]. We speculate that many African physicians may enter the UK at younger ages, often as students, and then decide to stay and practice in the UK. In contrast, African migrant physicians typically come to the US at comparatively older ages, to work or seek additional training after initial training and work in their home countries. Hence, despite the relatively larger population of SSA physicians and nurses in the UK, the transfer of medical skills from SSA to the US may be more significant than to the UK due to the increased level of education and professional experience that would-be migrants to the US possess at time of emigration.

Medical skill transfers from SSA result in remittances sent to the home countries by migrant physicians. In 2008, officially recorded remittances from all émigrés to Africa totaled US$41.1 billion, compared to US$39.4 billion of official development aid [33]. Also, a proportion of remittances unaccounted for, but believed to be significant, is sent through informal channels such as hand-delivery during short-term home visits [33]. In addition to remittances, some SSA medical migrants have reported sending medications, medical supplies, and diagnostic equipment to their home communities [34]. Nonetheless, while acknowledging the potential for remittances to support the livelihood of émigrés' relatives in origin countries, the large-scale emigration of medical professionals from poor to rich countries remains a serious drain on the health care workforce with both financial and human consequences. We state this assuming that émigré remittances are rarely invested in health care worker education, nor do they pay for physician salaries.

Mills and colleagues [35] estimated that nine SSA countries with HIV prevalence ≥5% have lost about US$2.2 billion of returns from the investment made on the medical education of their physicians who migrated to the UK, the US, Canada, and Australia. They also estimated that UK and the US have saved US$2.7 billion and US$846 million, respectively, from the services provided by these émigré physicians. *The World Health Report 2006* states: “Financial loss is not the most damaging outcome, however. When a country has a fragile health system, the loss of its workforce can bring the whole system close to collapse and the consequences can be measured in lives lost. In these circumstances, the calculus of international migration shifts from brain drain or gain to ‘fatal flows’” [2].

To characterize the magnitude of the SSA physician “brain drain” to the US, we examined a cross-section of SSA émigré physicians who successfully completed or were currently completing graduate medical education and specialty certification in the US (i.e., licensed and resident physicians; a resident is a physician training in a specialty, analogous to a registrar in the UK system). We use the phrase “SSA physician” to define any medical doctor born or trained in any countries located within the SSA sub-continent. We sought a rigorous recent count of émigré physicians trained in SSA and sub-Saharan African natives trained outside SSA who are now part of the physician workforce in the US, assessing both historical patterns and recent emigration trends.

## Methods

### Ethical Statement

An earlier draft of this paper was part of the first author's doctoral dissertation [34]. The work was approved by the Vanderbilt University Institutional Review Board. Prior to purchasing the dataset for the study, the first author also obtained approval from the American Medical Association for non-commercial use of physicians' data.

### Study Data

The American Medical Association Physician Masterfile (AMA-PM) was begun in 1906 to provide comprehensive biographic data on all US-based physicians. The AMA collects demographic, academic, and professional data on all residents and licensed physicians who practice in the US, including US medical graduates (USMGs) and IMGs. The annual data collection involves the voluntary cooperation of several health-related agencies, institutions, and organizations, including US medical schools, post-graduate medical training programs, state licensing agencies, the National Board of Medical Examiners (NBME), the ECFMG, and the American Board of Medical Specialties (ABMS) [36]. Quality issues in the AMA-PM records have been documented, namely undercounting, overcounting, and inaccurate addresses [37],[38]. For instance, the conflation of mailing/domicile and office addresses in the AMA-PM has yielded spatial errors in geocoding physician locations, resulting in overestimation of physician supply in certain affluent suburban neighborhoods where physicians reside but may not practice, and underestimation of supply in some inner city locations where physicians may practice but do not live [37]. These limitations notwithstanding, the AMA data are presently the best available representation of the US physician workforce, and are accessible to external users through AMA database licensees [39].

We obtained AMA data lists for all IMGs who received their education in medical schools located in SSA and all medical graduates who were born in SSA, but trained outside Africa. The records obtained included name, sex, medical school attended and year of graduation, year of birth, birth country, professional address, telephone number, primary specialty, residency program attended, and date of residency completion. The AMA does not collect information on residency admission date. We examined all records to identify and exclude potential outliers. Such outliers included 205 IMGs from three schools with uncertain location and/or legitimate existence: Kigezi International Medical School of Kabale (KIMSOK), St. Christopher's College of Medicine (SCCOM), and St. Luke School of Medicine (SLSOM). In our AMA dataset, these three schools were listed as located in Uganda, Senegal, and Liberia respectively.

However, KIMSOK (*n* = 51 IMGs) was actually located in the UK although accredited in Uganda until 2005, when its accreditation was revoked by Uganda's National Council for Higher Education [40]. SCCOM (*n* = 153 IMGs) claimed accreditation from Senegal's government, but had locations in Dakar, Senegal, and Luton, UK [41]. Side-by-side comparison of the surnames of all SCCOM graduates in our dataset with 263 popular surnames from 12 main Senegalese ethnic groups [42] produced no surname matches between the two groups; we believe that these SCCOM graduates were US nationals who trained in the Luton-based campus in the UK [43],[44]. Despite a report of multiple international locations in Liberia, Ghana, and California for SLSOM [41], we could merely identify a webpage for the school [45], suggesting that SLSOM may be essentially an internet-based training program. In 2005, SLSOM was disowned by Liberia's government as a ghost medical school, and removed from the International Medical Education Directory (IMED) at the request of Liberian authorities [41],[46],[47]. Only three IMGs in our data subset were trained in SLSOM. Birth countries of graduates from all three medical schools were missing for all but 21 IMGs in our dataset, only two of whom were born in SSA countries. We included these two IMGs in our analysis.

Since many older physicians are likely to have retired, we excluded those physicians >70 y (*n* = 442) for most analyses. We also excluded the five North African countries (Algeria, Egypt, Libya, Morocco, and Tunisia) since they have significantly different health care burdens, higher life expectancies, and higher physician-population ratios [5]. Information on country of birth and residency completion dates was incomplete. More specifically, there were 70.2% missing birth country and 10.5% missing residency completion dates among IMGs who graduated from SSA-based medical schools. Absence of birth country data especially limits our ability to analyze the extent of emigration among SSA-born but Western/overseas-trained physicians.

### Statistical Analysis

We used available data on residency completion dates to construct a proxy for year of immigration to the US by subtracting 5 y from SSA-IMGs' year of residency completion. Boulet et al. [48] analyzed trends in certification and residency training among IMGs and found that between 1995 and 2003, internal medicine, family practice, and pediatrics accounted for about 70% of IMGs specializations. Since residency training in the above primary care specializations is on average 3 y, we used 3 y as the minimum time spent in residency. We added 2 more years to the 3-y residency length in order to account for possible time spent in the US before the beginning of residency. The 2-y pre-residency length of stay in the US was estimated from primary data collected for a qualitative study of SSA-IMGs residing in the US [34].

From the comprehensive analysis of all SSA-IMGs appearing in the 2002 AMA-PM [19], we derived baseline metrics for our analysis, enabling calculation of the size of recent migration cohorts and the overall percentage increase of SSA-IMGs in the US during the last decade. We defined medical migration proportion (p) by the following formula: p = M÷(N+M) * 100, where M represents the number of SSA migrant physicians in the US, and N the stock of SSA physicians in the source country as reported by the WHO Global Health Workforce Statistics [5]. Likewise, we defined emigration percentage growth rate (r) as follows:
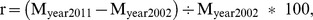
where M_year2011_ represents the number of SSA physicians in the 2011 AMA-PM, and M_year2002_ the number of SSA physicians in the 2002 AMA-PM. To estimate the length of service provided before immigration to the US among SSA-IMGs, we subtracted year of medical school graduation from the estimated year of entry in the US. Likewise, we subtracted the estimated age at entry in the US from migrant physicians' current age to estimate the length of time spent in the US. We used year of graduation as the chronological marker for the migration of SSA-born physicians trained outside SSA. We operationalized recent migrants as physicians who graduated or emigrated in 2000 or later. No SSA-IMGs from Cameroon, Tanzania, or Sudan were reported by Hagopian et al. [19], but appear in relatively significant numbers in our 2011 AMA-PM data subset. We used pre-and-post 2002 residency completion dates to estimate the emigration growth rate of Cameroonian, Tanzanian, and Sudanese émigré physicians.

To ascertain whether or not birth country information was missing completely at random, we used the Student's *t*-test to compare physicians with missing and complete birth country data on three variables: age, graduation age, and graduation year. We also used a series of scatter plots to highlight the linear relationship between the numerical distributions of variables among the main subgroups of migrant physicians identified in our analysis. We visualized temporal trends in physician graduation and emigration using histograms and smoothed lines. We used both Excel and the statistical package of social sciences (SPSS) to analyze the data.

## Results

**Figure 1:**
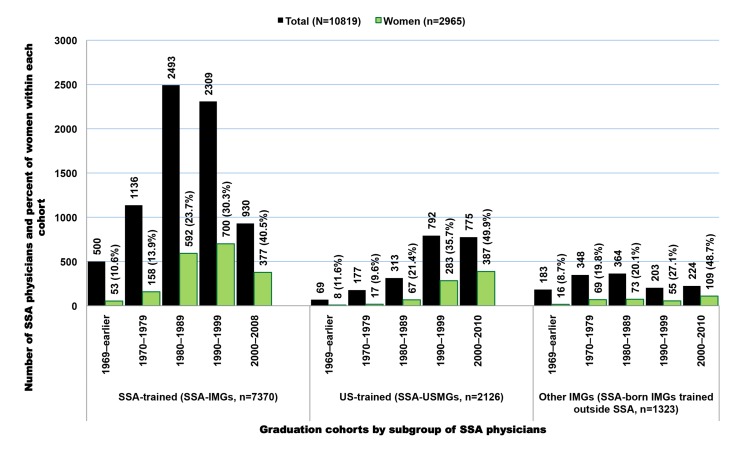
Subgroups of Sub-Saharan African migrant physicians identified in the US physician workforce. Data source: American Medical Association [115].

**Figure 2:**
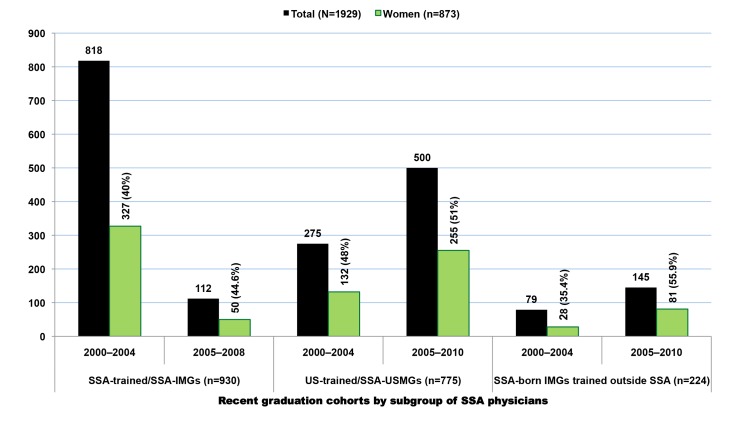
Sub-Saharan African migrant physicians who completed medical school in 2000 or later and are present in the US physician workforce. Data source: American Medical Association [115].

In the 2011 AMA-PM, 17,376 physicians were born or trained in Africa (Table 2). Physicians who were trained or born in the SSA subcontinent represent 62% (*n* = 10,819) of the total, with the remainder from North Africa (*n* = 6,557). Of the 10,819 SSA-origin physicians, 68% (*n* = 7,370) were trained in medical schools located in SSA (SSA-IMGs), 19.7% (*n* = 2,126) in US-based medical schools (SSA-USMGs), and 12.2% (*n* = 1,323) in medical schools located outside the SSA region and the US (Figure 1). About 18% (*n* = 1,929) of these émigrés can be considered early-career physicians or recent immigrants as they graduated in 2000 or later (Figure 2). As shown in both Figures 1 and 2, the proportional representation of women has consistently increased over time and across all three subgroups (SSA-IMGs, SSA-USMGs, and other IMGs).

**Table 2:** African-origin physicians appearing in the US physician workforce in 2011.

Sub-Regions	Main Regions of Medical Training				
	Africa	Americas	Asia and Pacific	Europe	Subtotal
**Sub-Saharan Africa**	7,370				7,370
**USA**		2,126			2,126
**India**			420		420
**Caribbean**		254			254
**Euro zone**				168	168
**UK**				154	154
**Middle East**			79		79
**Other**	28^a^	70	23	127	248
**Subtotal**	7,398	2,450	522	449	10,819
**North Africa**	6,557				6,557
**Total**	13,955	2,450	522	449	17,376

Data source: American Medical Association [115].

a These 28 physicians are sub-Saharan African-born IMGs trained in North Africa. They were included in our data analysis while the other 6,557 North African-trained and North African-born IMGs were excluded.

### SSA-Trained International Medical Graduates

**Figure 3:**
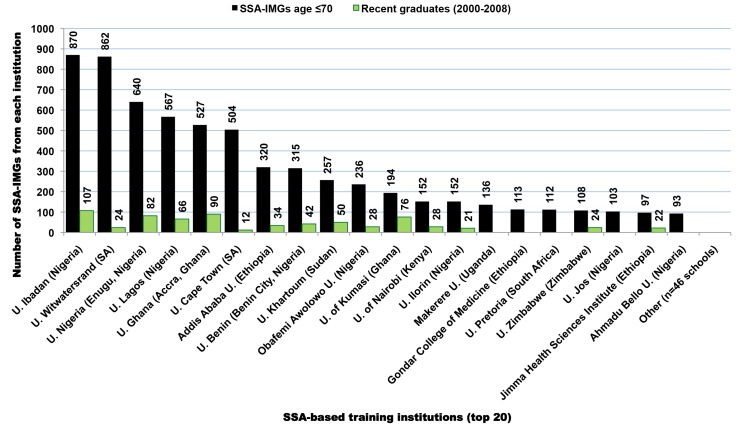
Sub-Saharan African-based institutions with the largest numbers of medical graduates appearing in the US physician workforce. Data source: American Medical Association [115].

SSA-IMGs (*n* = 7,370) appearing in the 2011 AMA-PM were trained in 28 SSA countries, with over two-third coming from medical schools in Nigeria and South Africa (Figure 3). The top 12 SSA countries of medical schools featured in our analysis represented 99% of SSA-IMGs in the US physician workforce (Table 3). The number of SSA-IMGs in the 2011 AMA-PM increased by over 38% (*n* = 2,036) since 2002, and over 50% (*n* = 1,113) of this increase came from Nigerian-trained IMGs (Table 4). However, Cameroon, Sudan, Ethiopia, and Kenya had the largest percentage increases of SSA-IMGs in the US physician workforce (AMA-PM) since 2002: 350% (*n* = 49), 287% (*n* = 244), 107% (*n* = 274), and 86% (*n* = 174), respectively. The number of IMGs from South Africa decreased by 8% (*n* = −157). In addition, of the 240 (3.3%) SSA-IMGs age >70 y excluded from most of our analysis, 87% (*n* = 209) were from South Africa, reflecting the seniority and early migration history of South African IMGs. Relative to the size of the physician workforce reported in the source countries, Liberian, Ghanaian, and Ethiopian-trained IMGs experienced the largest physician losses to the US, with 52.3% (*n* = 59), 26.2% (*n* = 721), and 22.7% (*n* = 531) of physicians emigrating respectively. This emigration proportion is based solely on the number of SSA-IMGs found in the 2011 AMA-PM. It would likely increase if one includes the sizeable numbers of SSA-born physicians trained in the US and SSA-born physicians trained in medical schools located outside the US and outside SSA. It will certainly be much higher if one includes all SSA migrant physicians in other main destination countries (e.g., the UK, Canada, or Australia).

**Table 3:** Sub-Saharan African-trained international medical graduates (SSA-IMGs) appearing in the US physician workforce in 2011.

Countries with ≥5 SSA-IMGs	SSA-IMGs		Potentially Active SSA-IMGs (age ≤70)		Recent Graduates (2000–2008)	
	*n*	Cumulative Percent	*n*	Cumulative Percent	*n*	Percent
Nigeria	3,271	44.4	3,252	45.6	433	13.3
South Africa	1,787	68.6	1,578	67.7	54	3.4
Ghana	721	78.4	721	77.9	166	23
Ethiopia	531	85.6	530	85.3	70	13.2
Sudan	329	90.1	328	89.9	80	24.4
Kenya	173	92.4	173	92.3	40	23.1
Uganda	145	94.4	139	94.3	16	11.5
Zimbabwe	112	95.9	108	95.8	24	22.2
Zambia	81	97	81	96.9	9	11.1
Cameroon	63	97.9	63	97.8	21	33.3
Liberia	56	98.6	56	98.6	0	0
Tanzania	24	99	24	98.9	2	8.3
Somalia	14	99.1	14	99.1	0	0
Congo (DRC)^a^	13	99.3	13	99.3	4	30.8
Senegal	11	99.5	11	99.5	3	27.3
Guinea	6	99.6	6	99.5	1	16.7
Sierra Leone	6	99.6	6	99.6	1	16.7
Ivory Coast	5	99.7	5	99.7	2	40
Other (*n* = 10)^a^	22	100	22	100	2	9.1
Total	7,370		7,130		928	13

Data source: American Medical Association [115].

a DRC is the Democratic Republic of Congo. Other includes ten countries with fewer than five SSA-IMGs each in the 2011 AMA Physician Masterfile.

**Table 4:** Change in the number of Sub-Saharan African-trained international medical graduates (SSA-IMGs) appearing in the US physician workforce between 2002 and 2011.

Countries of Training (Top 12)	2002 Data			2011 Data					Change	
	SSA-IMGs	Physicians in Source Countries	Emigration Fraction	SSA-IMGs	SSA-IMGs, age ≤70	Physicians in Source Countries	Emigration Fraction	“Active” Emigration Fraction	SSA-IMGs (n)	SSA-IMGs (%)
Cameroon^a^	[14]	[1,095]	1.3%	63	63	1,346	4.5%	4.5%	49	350.0%
Sudan^a^	[85]	[3,157]	2.6%	329	328	10,813	2.95%	2.9%	244	287.1%
Ethiopia	257	1,564	14.1%	531	530	2,152	19.8%	19.8%	274	106.6%
Kenya	93	4,001	2.3%	173	173	7,549	2.2%	2.2%	80	86.0%
Tanzania^a^	[14]	[1,384]	1.0%	24	24	300	7.4%	7.4%	10	71.4%
Nigeria	2,158	22,894	8.6%	3,271	3,252	55,376	5.6%	5.5%	1,113	51.6%
Ghana	478	1,210	28.3%	721	721	2,033	26.2%	26.2%	243	50.8%
Zimbabwe	75	1,694	4.2%	112	108	827	11.9%	11.6%	37	49.3%
Zambia	67	676	9.0%	81	81	836	8.8%	8.8%	14	20.9%
Liberia	47	72	39.5%	56	56	51	52.3%	52.3%	9	19.1%
Uganda	133	722	15.6%	145	139	3,361	4.1%	4.0%	12	9.0%
South Africa	1,943	23,844	7.5%	1,786	1,577	38,236	4.5%	4.0%	−157	−8.1%
Other^a^	83	12,912	0.6%	78	78	21,666	0.4%	0.4%	n/a	n/a
Total	5,334	69,589	7.1%	7,370	7,130	144,546	4.9%	4.7%	2,036	38.2%

Data sources: World Health Organization [5]; 2002 AMA Physician Masterfile as per Hagopian et al. [19]; American Medical Association [115].

a 2002 data were reported by Hagopian et al. [19] except for the numbers of IMGs trained in Cameroon, Tanzania, and Sudan. Their numbers are included in brackets because they are not part of the total counts reported in the last row of the table. These migrants were identified among SSA-IMGs in the 2011 AMA Physician Masterfile who completed residency by 2002. But the number of physicians available in Cameroon, Sudan, and Tanzania in 2002 came from the Hagopian et al. paper. In their dataset, “other” includes 12 countries with “at least one graduate in the US.” In our 2011 dataset, except otherwise specified, “other” refers to the 16 sub-Saharan African countries with fewer than 15 SSA-IMGs each in the 2011 AMA Physician Masterfile. The numbers of physicians in source countries for the year 2011 are from the Global Health Workforce Statistics of the World Health Organization [5]. “Active” emigration rate is the emigration rate among potentially active physicians. We defined all migrant physicians age ≤70 as potentially active.

### Demographic Characteristics of SSA-IMGs

Of the 7,130 potentially active migrant physicians (age ≤70) in our SSA-IMG data subset, 26% (*n* = 1,857) were women. The representation of women has consistently increased over time. From 10% in the earliest graduation cohort (1969 and earlier), women represent a little over 40% of all SSA-IMGs who graduated and emigrated during the last decade (Figures 1 and 2). As shown in Table 5, the mean year of birth of SSA-IMGs appearing in the 2011 AMA-PM is 1963 (standard deviation [SD] = 9.4), for a mean age of 50. On average, they completed medical school in 1988 (SD = 9.6) at age 25 (SD = 2.2), and then moved to the US about 6.5 y later. But, as reflected in the large standard deviations reported for each mean, these average figures vary substantially within and between source countries. With a mean age of 57 (SD = 9.4), South African SSA-IMGs are the oldest, and have been living in the US for over 26 y (SD = 10.3), the longest length of time of any groups. Sudanese migrants are the youngest (mean age = 45.1, SD = 8.0) and have been living in the US for about 13 y. Their age demographics and rapid migration are comparable to Cameroonian SSA-IMGs, the second youngest group from the nation with the fastest growing physician emigration rate.

**Table 5:** Birth, graduation, residency completion, and estimated US entry years among Sub-Saharan African-trained medical graduates (SSA-IMGs) appearing in the US physician workforce in 2011.

Countries of Training (Top 12)	SSA-IMGs age ≤70	Mean Year of Birth (SD)	Mean Year of Graduation (SD)	Mean Year of Residency Completion (SD)	Estimated Mean Year at US Entry (SD)	Mean Age (SD) in 2013	Mean Age (SD) at Graduation	Estimated Mean Age (SD) at US Entry	Mean Length of Time in US (SD)
South Africa	1,577	1956 (9.4)	1980 (9.4)	1991.8 (10.3)	1986.8 (10.3)	57 (9.4)	24.1 (1.6)	29.1 (4.7)	26.2 (10.3)
Uganda	139	1956.9 (12.4)	1982.1 (12.3)	1992.5 (13.4)	1987.5 (13.4)	56.2 (12.4)	25.3 (1.5)	29.8 (4.2)	25.5 (13.5)
Zambia	81	1960.6 (8)	1986.4 (7.9)	1995.6 (9.3)	1990.6 (9.3)	52.4 (8)	25.8 (2.2)	30.2 (4.4)	22.4 (9.4)
Tanzania	24	1959.6 (9.4)	1984.8 (9.3)	1997.6 (10.3)	1992.6 (10.3)	53.8 (9.4)	25.5 (1.3)	33 (6.0)	20.4 (10.6)
Liberia	56	1957 (4.6)	1984.7 (4.7)	1998.5 (6.7)	1993.5 (6.7)	56 (4.6)	27.7 (2.6)	36.4 (6.7)	19.5 (6.8)
Zimbabwe	108	1964.5 (10.2)	1989.2 (9.6)	2000.3 (9.5)	1995.3 (9.5)	48.6 (10.2)	24.8 (2.3)	29.8 (4.4)	17.7 (9.5)
Kenya	173	1964.6 (10)	1990.1 (10.4)	2000.4 (10.7)	1995.5 (10.7)	47.4 (10.2)	25.5 (1.2)	31.1 (5.1)	17.6 (10.8)
Ghana	721	1963.6 (9)	1990.7 (9.4)	2000.5 (8.8)	1995.5 (8.8)	49.4 (9)	27.1 (1.6)	31.8 (3.8)	17.5 (8.8)
Nigeria	3,252	1965.3 (7.6)	1990.2 (7.7)	2002.3 (7.3)	1997.3 (7.3)	47.7 (7.7)	24.8 (2.3)	31.9 (4.9)	15.7 (7.3)
Ethiopia	530	1965.8 (8)	1991 (7.6)	2004 (7.4)	1999.0 (7.4)	47.1 (8)	25.1 (1.9)	33.1 (4.7)	14.0 (7.4)
Cameroon	63	1967.2 (8.6)	1993.3 (8.5)	2005.2 (6.4)	2000.2 (6.4)	45.7 (8.6)	26.0 (1.4)	32.8 (4.6)	12.8 (6.5)
Sudan	328	1967.8 (7.9)	1993 (8)	2005.3 (6.2)	2000.3 (6.2)	45.1 (8)	25.1 (1.8)	32.7 (5.2)	12.7 (6.2)
Other (*n* = 16)^a^	78	1962.5 (7.7)	1990.6 (8.6)	2004.3 (3.8)	1999.3 (3.8)	50.6 (8.2)	28.1 (4.7)	36.2 (6.5)	13.7 (6.2)
Total	7,130	1962.9 (9.4)	1987.9 (9.6)	2000.1 (9.4)	1995.1 (9.4)	50.1 (9.4)	25.0 (2.2)	31.5 (5.0)	17.9 (9.4)

Data source: American Medical Association [115].

a Sub-Saharan African countries with <15 SSA-IMGs each in the 2011 AMA Physician Masterfile.

Note: Residency completion data were available for only 6,421 SSA-IMGs age ≤70. Given that the American Medical Association does not collect data on residency admission date, year of US entry was estimated by subtracting 5 y from residency completion year. Among SSA-IMGs still in residency at the time (*n* = 439), the 5-y lag was adjusted proportionally to the anticipated year of residency completion. Estimates were readjusted upward for those SSA-IMGs whose estimated year of entry in the US preceded their graduation year. For example, year of US entry for a Nigerian-trained physician who graduated from medical school in 2008 and was expected to complete residency in 2012, was readjusted from 2007 (i.e., 2012 minus 5 y) to 2008 (year of graduation). Estimates were readjusted downward for those SSA-IMGs whose estimated US entry year was above 2011.

### SSA-Based Medical Schools

SSA-IMGs appearing in the 2011 AMA-PM graduated from 66 SSA medical schools, with over 70% (*n* = 5,098) coming from only ten medical schools. Eighteen of 25 Nigerian medical schools are represented in the 2011 AMA-PM, with seven of them figuring in the top 20 (Figure 3). These seven Nigerian medical schools accounted for ≈39% (*n* = 2,883) of all SSA-IMGs and ≈90% of all Nigerian IMGs practicing in the US in 2011. Five of these schools are located in Southern Nigeria (Igboland and Yorubaland), and have >100 SSA-IMGs in the US each. Émigrés from medical schools located in Northern Nigeria (Hausaland), the largest geographic region of Nigeria, are comparatively underrepresented within the US-based Nigerian physician workforce. This may be due in part to Northern Nigeria having fewer medical schools (*n* = 6), and thus training fewer physicians than Southern Nigeria (*n* = 19 medical schools) [7].

Graduates from the University of Witwatersrand and the University of Cape Town make up >87% of all South African SSA-IMGs, but South African-trained migrant physicians represent fewer than 4% (*n* = 54) of recent SSA-IMGs (Table 3). Recent SSA-IMGs graduated between 2000 and 2008, the last year for which graduation records appear among SSA-IMGs in the 2011 AMA-PM, and they attended 56 SSA-based medical schools. Respectively, 46.5% (*n* = 433), 18% (*n* = 166), and 9% (*n* = 80) of these recent graduates come from Nigerian, Ghanaian, and Sudanese institutions. As shown in Figure 3, the University of Ibadan (*n* = 107; Nigeria), the University of Accra (*n* = 90; Ghana), the University of Nigeria in Enugu (*n* = 82; Nigeria), the University of Kumasi (*n* = 76; Ghana), and the University of Lagos (*n* = 66; Nigeria) are the top five medical schools with the largest numbers of recent SSA-IMGs in the 2011 AMA-PM.

With 28 medical schools, Sudan has the largest number of medical schools on the African continent [7]. While the 2000–2008 Sudanese graduates account for only 24.3% (*n* = 80) of the total Sudanese SSA-IMGs in the 2011 AMA-PM, 244 Sudanese SSA-IMGs have actually been added to the AMA-PM since 2002. Nearly 80% (*n* = 258) of all Sudanese SSA-IMGs present in the 2011 AMA-PM graduated from the University of Khartoum. Post-2002 residency completions among graduates from the University of Khartoum represent 75% (*n* = 163) of 216 Sudanese SSA-IMGs completing residency after 2002.

### Residency Institutions and Primary Specialty

Over 600 US-based residency programs provided post-graduate training to 6,517 SSA-IMGs with complete residency records in the 2011 AMA-PM. Howard University Hospital was the most popular of these residency institutions, and trained ≈5% (*n* = 314) of those SSA-IMGs with complete residency training information. Four of five residency programs that recruited ≥100 SSA-IMGs are located in New York City and its suburbs (Table 6). With 3,347 SSA-IMGs, the primary care specialties of internal medicine, pediatrics, and family practice represent ≈50% of all identified medical and surgical specialties (Table 7). This percentage would be higher if we included the various subspecialties within these primary specialties. While women represented less than one-third of the overall SSA-IMG population, they outnumbered men in pediatrics. Surgical specializations were uncommon among SSA-IMGs, representing <2% (*n* = 143) of all SSA-IMGs with identified specialties (*n* = 7,298).

**Table 6:** US residency institutions that trained the highest number of Sub-Saharan African physicians appearing in the US physician workforce in 2011.

Top 20 Residency Programs	SSA-IMGs^a^		SSA-USMGs^a^		Other IMGs^a^		Total	
	*n*	Women (%)	*n*	Women (%)	*n*	Women (%)	*n*	Women (%)
Howard University Hospital (Washington, D.C.)	315	87 (27.6%)	51	6 (11.8%)	22	2 (9.1%)	388	95 (24.5%)
Harlem Hospital Center (New York, NY)	273	52 (19%)	14	2 (14.3%)	10	1 (10%)	298	55 (18.5%)
Mt Sinai School of Medicine (New York, NY)	164	41 (25%)	11	4 (36.4%)	13	2 (15.4%)	188	47 (25%)
New York Medical College (Valhalla, NY)	137	33 (24.1%)	20	11 (55%)	21	8 (38.1%)	178	52 (29.2%)
John H. Stroger Jr. Hospital of Cook County (Chicago, IL)	133	47 (33.3%)	11	2 (18.2%)	13	2 (15.4%)	157	51 (32.5%)
Bronx-Lebanon Hospital Center (Bronx, NY)	100	26 (26%)	4	3 (75%)	12	2 (16.7%)	116	31 (26.7%)
Wayne State University (Detroit, MI)	66	20 (30.3%)	22	9 (40.9%)	23	3 (13%)	111	30 (27%)
Johns Hopkins University (Baltimore, MD)	50	3 (6%)	47	21 (44.7%)	9	1 (11.1%)	107	25 (23.4)
SUNY Health Sciences Center (Brooklyn, NY)	91	18 (19.8%)	9	3 (33.3%)	7	3 (42.9%)	107	24 (22.4%)
University of Maryland Medical System (Baltimore, MD)	71	23 (32.4%)	27	13 (48.1%)	3	1 (33.3%)	101	37 (36.6%)
Mayo Grad. School of Med./Mayo Fndn. (Rochester, MN)	63	7 (11.1%)	22	5 (22.7%)	13	2 (15.4%)	98	14 (14.3%)
Baylor College of Medicine (Dallas, TX)	59	19 (32.2%)	28	7 (25%)	10	3 (30%)	97	29 (29.9%)
Emory University School of Medicine (Atlanta, GA)	60	16 (26.7%)	35	17 (48.6)	2	0	97	33 (34%)
A Einstein College of Medicine of Yeshiva U. (New York, NY)	70	19 (27.1%)	18	11 (61.1%)	2	0	90	30 (33.3%)
Morehouse School of Medicine (Atlanta, GA)	74	36 (48.6%)	5	4 (80%)	6	1 (16.7%)	85	41 (48.2%)
Interfaith Medical Center (Brooklyn, NY)	73	9 (12.3%)	4	1 (25%)	5	0	82	10 (12.2%)
Henry Ford Hospital (Detroit, MI)	40	20 (50%)	21	5 (23.8%)	10	3 (30%)	71	28 (39.4%)
Woodhull Medical & Mental Health Center (Brooklyn, NY)	69	22 (31.9%)	0	0	2	1 (50%)	71	23 (32.4%)
Metrohealth Medical Center (Cleveland, OH)	54	16 (29.6%)	10	3 (30%)	5	2 (40%)	69	21 (30.4%)
Jackson Memorial Hospital/Jackson Health Services (Miami, FL)	39	10 (25.6%)	21	10 (47.6%)	7	1 (14.3%)	67	21 (31.3%)
Subtotal	2,001	524 (26.2%)	380	137 (36%)	195	38 (19.5%)	2,578	697 (27%)
Other *(n*>800 residency programs)	4,562	1,157 (25.4%)	1,688	591 (35%)	990	225 (22.7%)	7,238	1,990 (27.5%)
Total records with residency program information	6,563	33.5%)	2,068	729 (35.2)	1,185	263 (26.6%)	9,816	2,687 (27.4%)

Data source: American Medical Association [115].

a SSA-IMGs, sub-Saharan African-trained medical graduates; SSA-USMGs, US-trained medical graduates born in sub-Saharan Africa; Other IMGs, sub-Saharan African-born internationals medical graduates trained outside sub-Saharan Africa.

**Table 7:** Primary specialty choices among Sub-Saharan African migrant physicians appearing in the US physician workforce in 2011.

Top 20 Primary Specialties	SSA-IMGs^a^		SSA-USMGs^a^		Other IMGs^a^		Total	
	*n*	Women (%)	*n*	Women (%)	*n*	Women (%)	*n*	Women (%)
Internal medicine	2,037	523 (25.7%)	362	151 (41.7%)	255	73 (28.6%)	2,654	747 (28.1%)
Family medicine	648	248 (38.3%)	193	80 (41.5%)	161	63 (39.1%)	1,002	391 (39%)
Pediatrics	662	343 (51.8%)	105	79 (75.2%)	82	41 (50%)	849	463 (54.5%)
Psychiatry	345	89 (25%)	44	22 (50%)	75	16 (21.3%)	464	127 (27.4%)
Obstetrics and gynecology	208	39 (18.8%)	207	104 (50.2%)	37	10 (27%)	452	153 (33.8%)
Anesthesiology	275	50 (18.2%)	99	30 (30.3%)	58	12 (20.7%)	432	92 (21.3%)
General surgery	143	4 (2.8%)	130	29 (22.3%)	35	2 (5.7%)	308	35 (11.4%)
Cardiovascular disease	194	12 (6.2%)	57	10 (17.5%)	55	6 (10.9%)	306	28 (9.2%)
Nephrology	157	24 (15.3%)	35	13 (37.1%)	24	3 (12.5%)	216	40 (18.5%)
Diagnostic radiology	118	18 (15.3%)	46	11 (23.9%)	26	5 (19.2%)	190	34 (17.9%)
Infectious disease	137	30 (21.9%)	30	20 (66.7%)	18	5 (27.8%)	185	55 (29.7%)
Gastroenterology	124	8 (6.5%)	24	5 (20.8%)	27	2 (7.4%)	175	15 (8.6%)
Emergency medicine	50	3 (6%)	102	32 (31.4%)	16	2 (12.5%)	168	37 (22%)
Neurology	86	11 (12.8%)	23	7 (30.4%)	32	4 (12.5%)	141	22 (15.6%)
Neonatal-perinatal medicine	114	36 (31.6%)	9	6 (66.7%)	12	2 (16.7%)	135	44 (32.6%)
Anatomic/clinical pathology	96	33 (34.4%)	16	4 (25%)	15	8 (53.3%)	127	45 (35%)
Pulmonary critical care medicine	92	4 (4.3%)	19	7 (36.8%)	7	2 (28.6%)	118	13 (11%)
Ophthalmology	41	7 (17.1%)	52	19 (36.5%)	12	0	105	26 (24.8%)
Hematology/oncology	79	16 (20.3%)	9	3 (33.3%)	13	4 (30%)	101	23 (22.8%)
Endocrinology diabetes and metabolism	78	19 (24.4%)	14	8 (57.1%)	8	3 (37.5%)	100	30 (30%)
Subtotal	5,684	1,517 (26.7%)	1,576	640 (40.6%)	968	263 (27.2%)	8,228	2,420 (29.4%)
Other (*n*>130 specialties and sub-specialties)	1,204	266 (22.1%)	439	106 (24.2%)	208	48 (23.1%)	1,851	421 (22.7%)
Total records with specialty information	6,888	1,786 (25.9%)	2,015	746 (37%)	1,176	311 (26.4%)	10,079	2,841 (28.2%)

Data source: American Medical Association [115].

a SSA-IMGs, sub-Saharan African-trained medical graduates; SSA-USMGs, US-trained medical graduates born in sub-Saharan Africa; Other IMGs, sub-Saharan African-born internationals medical graduates trained outside sub-Saharan Africa.

### Migration Cohorts

**Figure 4:**
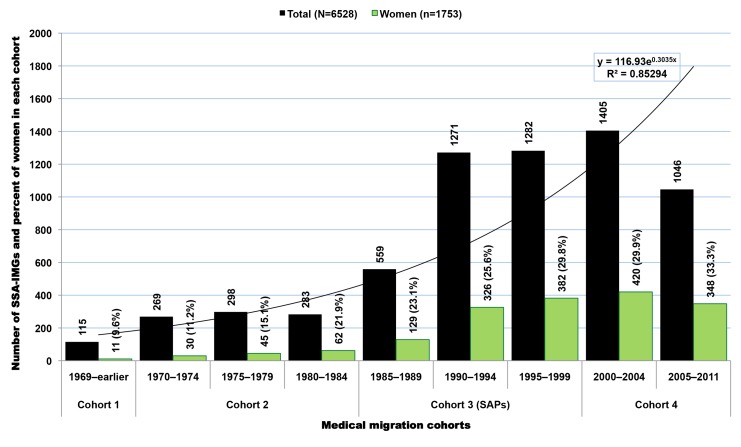
Growth over time of migration among Sub-Saharan African-trained medical graduates appearing in the US physician workforce. Data source: American Medical Association [115]. As reflected by the exponential equation and the determination coefficient (R^2^) of the smoothed line displayed on the chart, the distribution of the data approaches an exponential curve, and reflects the rapid emigration growth of sub-Saharan African trained medical graduates. The increase in emigration is particularly significant in cohort 3, which mainly coincides with the implementation period of the SAPs.

Available residency data on SSA-IMGs indicate that the earliest cohort of SSA-IMGs entering the US (cohort 1) was composed primarily of graduates of South African medical schools, Makerere University in Uganda, and the University of Ibadan in Nigeria. These first émigrés came to the US prior to the 1970s, when African medical schools were smaller, fewer in number, and heavily reliant on the expertise of expatriates from former colonial powers [49],[50]. The second cohort (cohort 2) reflects SSA-IMGs who emigrated in the 1970s and early 1980s, a time when most SSA countries opened at least one medical school and graduated at least the first domestic batch of MD-equivalent Bachelor of Medicine, Bachelor of Surgery (MBChB or MBBS) graduates [8],[9]. Except in apartheid-plagued South Africa, opportunities for further medical specialization were largely unavailable to SSA-IMGs graduating in this period. Cohorts 1 and 2 are smaller than subsequent cohorts and represent 14.8% (*n* = 965) of SSA-IMGs with available residency information in the 2011 AMA-PM (Figure 4); physician retirements and deaths are to be expected in these older cohorts. Interestingly, South African IMGs, most of whom are white, represent 60% (*n* = 578) of these early SSA migrants. By and large, they were escaping apartheid [51], the legally sanctioned racist policy governing South Africa from the late 1940s to early 1990s.

The third migration cohort (cohort 3) represents 47.7% (*n* = 3,112) of SSA-IMGs. They came to the US between the mid-1980s and late 1990s. The surge in medical migration unfolding in the mid-1980s and sharply increasing throughout the following decade (1991–2000) coincided with the implementation of austere economic measures in low- and middle-income countries (LMICs), resulting in shrinkage of the public sector in African countries, termed structural adjustment programs (SAPs) [52]–[54]. The last migration cohort (cohort 4) comprises SSA-IMGs who came to the US during the new millennium, representing 37.5% (*n* = 2,451) of the US-based SSA-IMGs population; 31% (*n* = 758) among them were still in residency or had just completed their residency training in 2011. Of note, the emigration trend line in Figure 4 has the feature of an exponential growth curve, and the drop in the number of émigrés observed between 2005 and 2011 is likely to be an artifact of incomplete residency data, given lags in entry of recent data.

### Proxy for Years of Service Provided by SSA-IMGs Prior to Emigration

**Figure 5:**
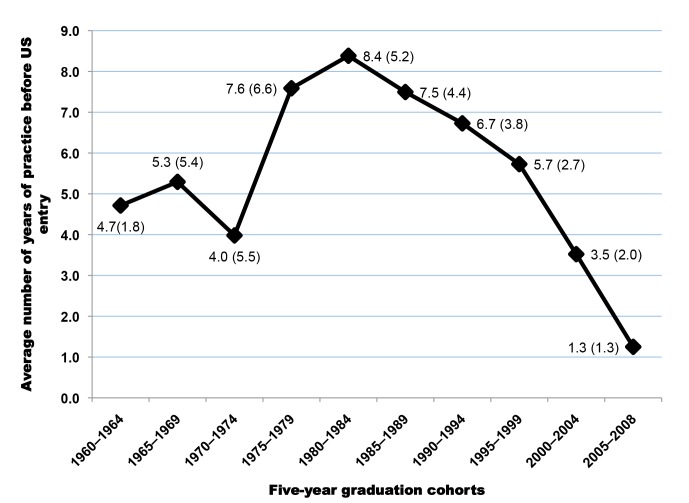
Length of service provided to the home country by each graduation cohort of Sub-Saharan African-trained medical graduates before migration to the United States. Note: Based on n = 6,421 complete graduation and residency records. Estimated mean year of time between graduation and entry in the United States: 6.4 y (SD = 4.6). Data source: American Medical Association [115].

Figure 5 illustrates the differences between the average year of entry in the US and the average year of graduation in SSA for each 5-y graduation cohort of SSA-IMGs. The plotted differences are proxies for the average number of years of service provided to the home countries by each cohort prior to emigration; this assumes that émigrés did not spend time in a third country prior to coming to the US. The steady decline observed among graduating cohorts beginning in the mid 1980s suggests that since the onset of the SAPs, SSA-IMGs have provided fewer years of service over time to their native countries before emigrating. SSA-IMGs who graduated between 2000 and 2008 may have practiced on average for 2.4 y before leaving. This suggests that many among them may have stayed in their native countries just long enough to complete the compulsory work for full graduation/licensure (typically a 1-y housemanship/internship and 1-y national service).

### SSA-IMGs with Birth Country Data

While physicians reporting to the AMA are required to report the schools where they obtained their training, the reporting of country of birth is not deemed mandatory; birth country information among SSA-trained IMGs was available for only 29.8% (*n* = 2,199) in the 2011 AMA-PM. Mean differences between SSA-IMGs with complete and missing birth country data were statistically significant at the 0.05 level for migrants' current age and graduation age, but not for graduation year, suggesting that an assumption that data are missing completely at random is not tenable (Tables S1 and S2). However, while we cannot confirm that the birth country data were missing completely at random, we found very high or near perfect correlations between both groups on the distribution of several variables of interest: age (R^2^ = 0.84), graduating age (R^2^ = 0.99), emigration age (R^2^ = 0.97), and primary specialty choice (R^2^ = 0.98) (Figures S1, S2, S3, S4). Thus, whatever statistical difference may exist between the two groups is not likely to affect inferences.

**Figure 6:**
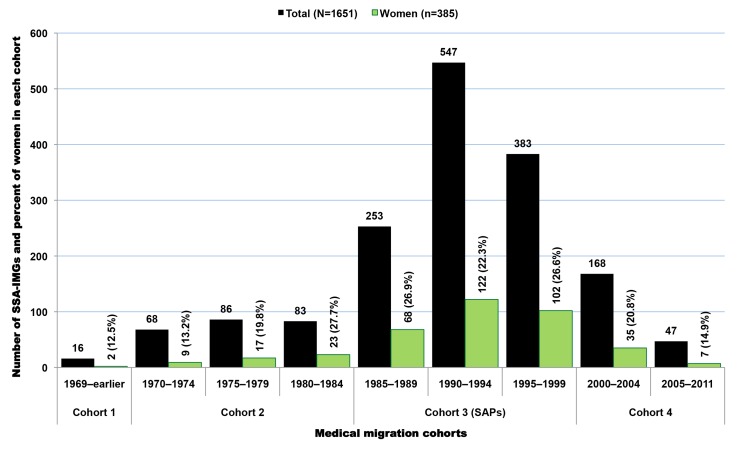
Emigration trends among Sub-Saharan African-trained medical graduates with complete birth country data in the 2011 AMA Physician Masterfile. Data source: American Medical Association [115].

**Figure 7:**
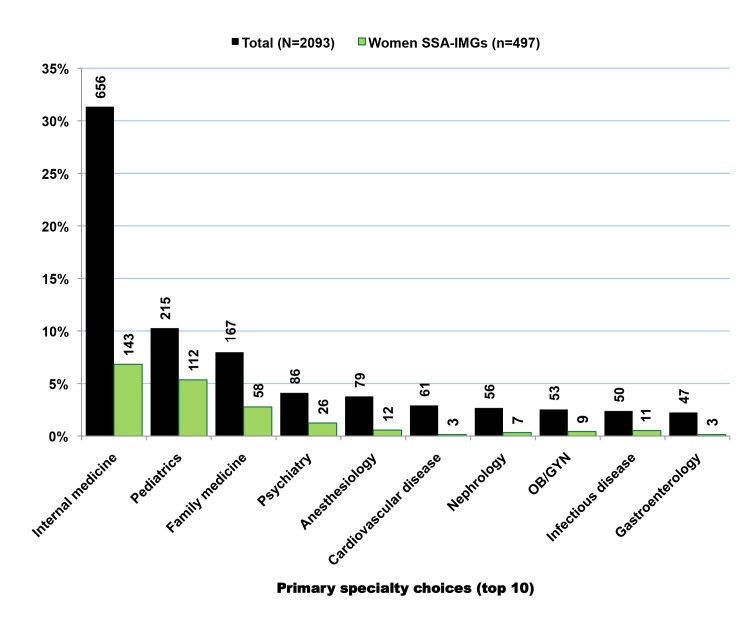
Trends in primary specialty among Sub-Saharan African-trained medical graduates with complete birth country data in the 2011 AMA Physician Masterfile. Data source: American Medical Association [115].

Of SSA-trained physicians in the US with complete birth country data, 21% (*n* = 467) attended medical schools outside their birth countries, and 15.6% (*n* = 342) were born in 34 non-African countries, led by the US, UK, India, and South Korea (Table S3). Nigeria and South Africa trained many students from other neighboring African countries who then migrated to the west, including the US. Of 752 Nigerian-trained IMGs, 26.5% (*n* = 199) reported a birth country other than Nigeria. Likewise, 41.5% (*n* = 156) of South African-trained IMGs were born outside of South Africa. This fraction is the highest for Zambia (65.2%, *n* = 30); the majority of Zambian-trained IMGs practicing in the US were born in India. In contrast, the vast majority of IMGs trained in Cameroon, Ethiopia, Ghana, Kenya, and Sudan are natives of those countries. Further, 24% (*n* = 510) of SSA-IMGs with complete birth country data are women. Of those with residency completion information (*n* = 1,651), over 70% (*n* = 1,183) immigrated to the US during the implementation years of the SAPs, and they represent 38% of all SSA-IMGs who moved to the US during that time-period (Figure 6). The trend in specialty choices among these émigrés with complete birth country data is quite similar to that of their counterparts with missing birth country data, with 49.6% (*n* = 1,038) specializing in primary care (internal medicine, pediatrics, and family medicine), and women outnumbering men in pediatrics (Figure 7). In sum, the 30% of physicians with complete birth country data among SSA-IMGs appear to be a fairly representative sample of the entire SSA-trained physician population in the 2011 AMA-PM.

### SSA-Born US Medical Graduates

**Figure 8:**
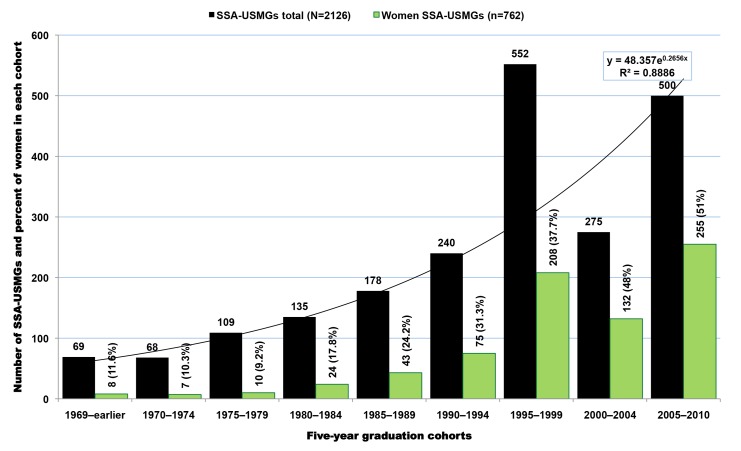
Graduation trends among Sub-Saharan African-born graduates of medical schools located in the United States. Data source: American Medical Association [115]. As reflected by the exponential equation and the determination coefficient (R^2^) of the smoothed line displayed on the chart, the distribution of the data approaches an exponential curve, reflecting a rapid numerical increase of sub-Saharan African natives graduating from medical school in the US.

In addition to SSA-trained physicians, there are 2,126 SSA-born migrant physicians who graduated from US-based medical schools in the 2011 AMA-PM. A small number of them (*n* = 73, 3.4%) are older than 70 y old and may likely be retired. When excluding the latter, the average SSA-born USMG is 46.3 y old (SD = 10.5), and graduated from medical school at age 29.3 (SD = 3.9), or 4 y later than the average SSA-IMG (Table 8). This graduation age gap between the two groups is intuitively understandable given that USMGs typically complete a 4-y undergraduate degree program before admission to medical school, whereas their counterparts in SSA enter medical school directly from high school. Cameroonian-born SSA-USMGs are the youngest, with a mean age of 40.7 y (SD = 10.7), while Tanzanian-born are the oldest (mean age = 56.3, SD = 7.7). With a mean age of 42.2 y (SD = 8.6) and a mean graduation age of 28.5 y (SD = 3.4), women SSA-USMGs are 6 y younger than men (mean age = 48.8; SD = 10.7), and they graduated 1.2 y earlier than their male counterparts. Overall, women represent 36% (*n* = 762) of this subgroup of SSA-born physicians educated in the US. However, if the current trends persist, their number will proportionately increase as suggested by current trends and their majority status among African physician graduates between 2005 and 2010 (Figure 8).

**Table 8:** Sub-Saharan African-born physicians who graduated from US-based medical schools (SSA-USMGs).

Birth Country	SSA-USMGs Age ≤70			Women SSA-USMGs Age ≤70			Graduated in 2000 and Later	
	*n*	Mean Age (SD) in 2013	Mean Age (SD) at Graduation	*n*	Mean Age (SD) in 2013	Mean Age (SD) at Graduation	*n*	Women (%)
Ghana	398	44.4 (10.5)	30.4 (4.3)	152	40.0 (7.5)	28.9 (3.1)	216	101 (46.8%)
Nigeria	384	56 (9)	30.4 (4.4)	84	46.7 (9.0)	29.0 (4.3)	18	10 (55.6%)
Kenya	292	42 (8.3)	28.3 (3.1)	111	40.5 (7.8)	28.6 (3.3)	162	71 (44%)
Ethiopia	235	44.5 (8.6)	29.6 (3.9)	110	41.3 (6.8)	29.0 (3.7)	96	58 (60.4%)
South Africa	174	46.4 (6.4)	27.9 (2.9)	59	46.3 (6.5)	27.8 (3.4)	12	4 (33.3%)
Liberia	92	43.2 (9.6)	28.6 (3.3)	50	40.6 (8.4)	27.9 (3.1)	46	29 (63%)
Zambia	82	39 (7.2)	26.6 (2)	32	38.0 (6.6)	26.8 (2.2)	44	20 (45.5%)
Uganda	69	46.6 (7.6)	29 (4.6)	21	43.4 (5.4)	27.9 (4.4)	18	4 (22.2%)
Cameroon	66	40.7 (10.7)	29.4 (3.5)	30	35.4 (5.1)	29.1 (3.7)	51	29 (57%)
Zimbabwe	40	52.3 (8)	28.1 (3.1)	14	50.2 (8.6)	27.6 (2.2)	1	1 (100%)
Tanzania	35	56.3 (6.6)	27.6 (3.1)	9	54.6 (6.1)	27.9 (3.3)	0	0
Sudan	31	41.6 (10.7)	28.4 (2.9)	17	36.5 (4.7)	27.9 (2.9)	24	16 (66.7%)
Other (*n* = 24)^a^	155	43.9 (10.8)	29.2 (3.8)	66	40.8 (9.2)	28.3 (2.7)	87	44 (50.6%)
Total	2,053	46.3 (10.5)	29.3 (3.9)	755	42.2 (8.6)	28.5 (3.4)	775	387 (49.9%)

Data source: American Medical Association [115].

a Each of these 24 sub-Saharan African countries had <20 USMGs, except Somalia which had 32 USMGs but had <15 SSA-IMGs in the 2011 AMA Physician Masterfile, and as such was not included among the top 12 sub-Saharan African source countries.

Among SSA countries with US-based physicians ≤70 y old, Ghana, a country with 27 times fewer medical doctors than Nigeria (see e.g., Table 1), has the highest absolute number of US-trained physicians in the US (Table 8). As was the case with SSA-trained migrant physicians, most of the same leading countries appear in the top five for SSA-USMGs: Ghana (*n* = 398), Nigeria (*n* = 383), Kenya (*n* = 292), Ethiopia (*n* = 235), and South Africa (*n* = 174). Ghanaian and Kenyan-born USMGs represent, respectively, 27.9% (*n* = 216) and 21.3% (*n* = 162) of all SSA-USMGs who obtained their medical degrees in the US between 2000 and 2010. The number of SSA-USMGs in the AMA-PM appears to determine in part the number of SSA-trained IMGs and the number of SSA-born IMGs trained outside SSA found in the AMA-PM. The scatter plots (Figures S5, S6, S7) of the bivariate relationships between the numbers of USMGs and IMGs yielded the following determination coefficients: R^2^ = 0.5 (for SSA-USMGs and SSA-IMGs), R^2^ = 0.49 (for SSA-USMGs and SSA-born IMGs trained outside SSA), and R^2^ = 0.08 (for SSA-IMGs and SSA-born IMGs trained outside SSA). While the last value is low and reflects the weak influence of both groups of IMGs on each other, the first two coefficients are quite high, and suggest a strong influence of the number of SSA-USMGs on both the number of SSA-IMGs and the number of other SSA-born IMGs trained outside SSA in the AMA-PM.

The graduation trend among SSA-USMGs (Figure 8) is strikingly similar to the emigration pattern observed among SSA-IMGs (Figure 4). Overall, the number of migrants in both groups has increased fairly consistently over time, except in the first half of 2000–2010, when one can observe a sharp decrease in the number of SSA-USMGs. After this post-September 11, 2001 (the date of the World Trade Center terrorism attack) downward trend, a surge in graduation is seen, with the greatest increase (51%, *n* = 255) occurring among women SSA-USMGs. The graduation trend line among SSA-USMGs also approaches an exponential growth curve. This may reflect a recent fast pace of emigration among SSA natives pursuing medical education in the US. This may equally reflect the increasing gain over time realized by the US physician workforce as the immigrant children of SSA-IMGs who are raised in the US are increasingly entering, and graduating from, US medical schools.

### US-Based Medical Schools, Residency Programs, and Primary Specialties

**Figure 9:**
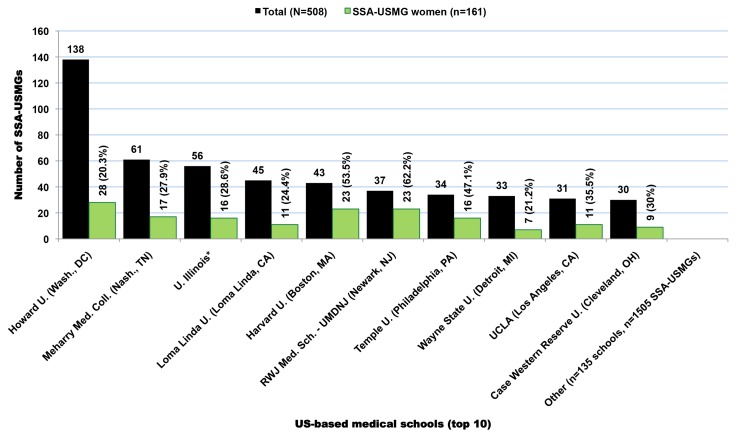
American medical schools with the highest number of Sub-Saharan African-born graduates practicing in the United States. *Note: No information was provided as to which campus of the University of Illinois these sub-Saharan African-born medical graduates attended. Data source: American Medical Association [115].

The 2,126 SSA-USMGs identified in the 2011 AMA-PM attended 139 US-based medical schools. The ten medical schools that graduated the highest numbers of SSA-USMGs are highlighted in Figure 9. Howard University, arguably the most famous historically black college and university, is the most popular medical school among SSA-USMGs, and the only medical school to graduate >100 SSA-USMGs. Meharry Medical College is another historically black college, and it ranked second in graduation of SSA-USMGs (Figure 9). Likewise, 2068 SSA-USMGs attended >400 US residency programs, and Howard University Hospital trained the highest number (*n* = 51), with Harlem Hospital Center ranked second (Table 6). Fewer than one-quarter of the SSA-USMGs that Howard University trained in both its medical school (*n* = 28) and its residency program (*n* = 6) were women. Among physicians age ≤70 y, Johns Hopkins University School of Medicine has trained over three times more SSA-USMG women (*n* = 21) in its residency program than has Howard University Hospital.

Internal medicine is the most popular specialty choice among SSA migrant physicians, including SSA-USMGs (Table 7). Among the latter, it represents 18% (*n* = 362 physicians) of all primary specialty choices (n>90). Among the top 20 specialties reported in Table 7, SSA-USMG women outnumber men in pediatrics (75%, *n* = 79), obstetrics and gynecology (50.2%, *n* = 104), infectious disease (66.7%, *n* = 20), neonatal-perinatal medicine (66.7%, *n* = 6), and endocrinology diabetes and metabolism (57.1%, *n* = 8). Of the top 20 specialties reported, emergency medicine and ophthalmology are the only two fields of specialization with more SSA-USMGs than SSA-IMGs, with respectively 102 and 50 SSA-USMGs, compared to 50 and 41 SSA-IMGs.

### SSA-Born IMGs Trained outside SSA

**Figure 10:**
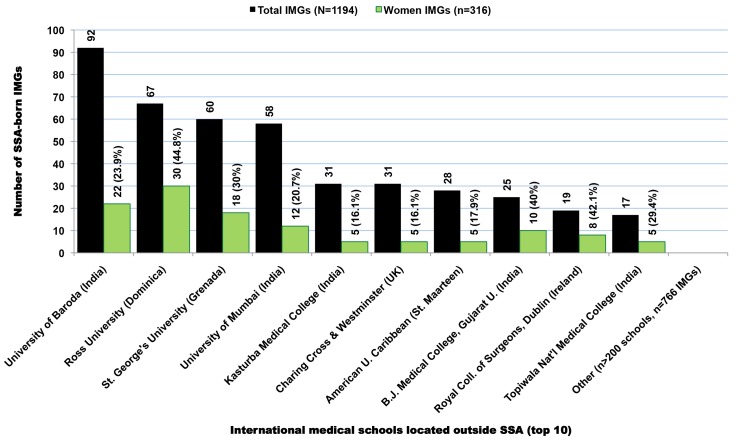
Foreign medical schools located outside Sub-Saharan Africa with the highest number of Sub-Saharan African-born medical graduates practicing in the United States. Data source: American Medical Association [115].

**Figure 11:**
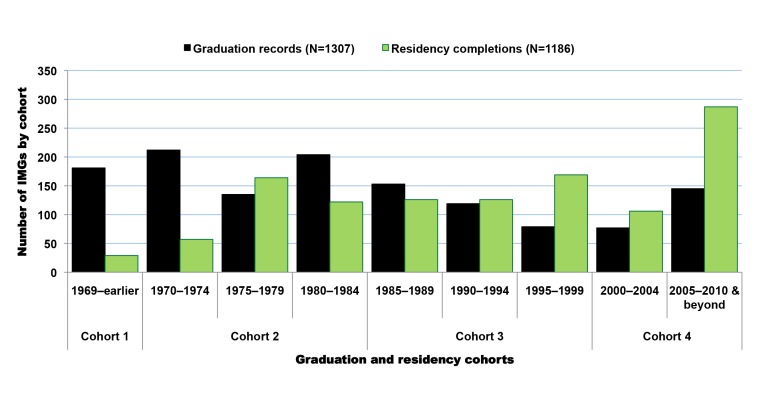
Graduation and residency trends among Sub-Saharan African-born international medical graduates educated outside Sub-Saharan Africa. Data source: American Medical Association [115].

**Figure 12:**
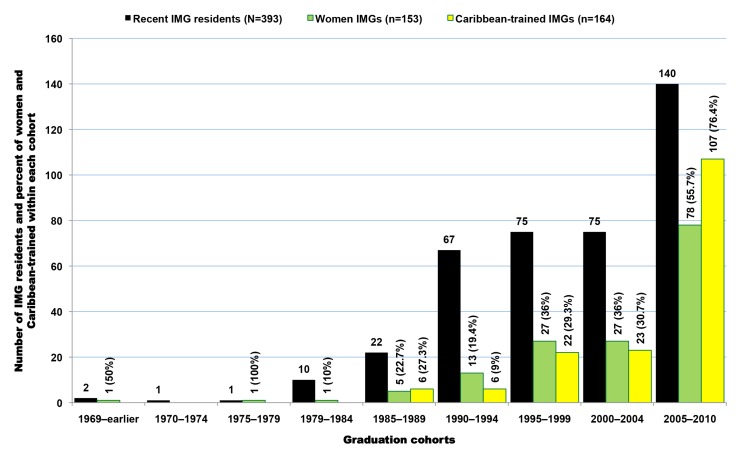
Recent demographic trends among Sub-Saharan African-born international medical graduates educated outside Sub-Saharan Africa and completing residency in the United States in 2000 or later. Data source: American Medical Association [115].

In the 2011 AMA-PM, there are 1,323 SSA-born physicians who were trained in medical schools located outside both SSA and the US (Figure 10). When excluding the nearly 10% (*n* = 129) of all senior and potentially retired physicians age >70, these SSA-origin IMGs are still the oldest group (mean age = 53.1; SD = 11.1), and the most heterogeneous, having been trained in >200 medical schools located in 69 foreign countries outside the SSA subcontinent and the US. While Nigeria (*n* = 127) and Ghana (*n* = 107) still appear among the leading countries in this group, 47% (*n* = 560) of these IMGs were born in the Southeastern region of Africa, namely in Kenya (*n* = 365), Uganda (*n* = 113), and Tanzania (*n* = 82) (Table 9). Many among them are likely of Indian ancestry. A little over 31% (*n* = 380) of these émigrés were educated in India, 21.2% (*n* = 253) in the Caribbean, and 11% (*n* = 132) in the UK. As illustrated in Figure 11, between the mid-1970s and the late 1990s the residency completion trend among IMGs is relatively flat, while graduations decrease gradually from early 1980s to mid-2000. Starting in 2005, both graduation and residency records are on the increase with a significant surge in residency admissions and completions. This latest increase in residents may be due in part to the growing number of US-based immigrants from SSA who do not gain entrée into US medical schools, but complete their medical educations in the Caribbean. Upon completion of medical school overseas, they typically return to the US to complete their residency and practice. As revealed by Figure 12, these Caribbean-trained IMGs represent >75% of all SSA-born IMGs trained outside SSA admitted into residency in the US after 2004.

**Table 9:** Sub-Saharan African-born international medical graduates who graduated from schools outside Sub-Saharan Africa.

Birth Country	IMGs Age ≤70					Graduated in 2000 and Later		
	*n*	Percent	Mean Age (SD) in 2013	Mean Age (SD) at Graduation	Mean Age (SD) at Residency Completion	*n*	Mean Age (SD) at Graduation	Women (%)
Kenya	365	30.6%	57.7 (10.1)	25.7 (3)	36.2 (9.2)	37	29.3 (6.0)	19 (51.4%)
Nigeria	127	10.6%	56.5 (7)	28.5 (4.20	38.4 (8.6)	0		0
Uganda	113	9.5%	57.9 (10.1)	25.8 (2.4)	37.1 (10.1)	8	27.1 (1.7)	7 (87.5%)
Ghana	107	9%	48.8 (11.1)	29.7 (3.7)	38.5 (7.6)	37	30.6 (4.5)	19 (51.4%)
Tanzania	82	6.9%	61.7 (7.7)	25.6 (2.3)	37.7 (10.9)	0		0
Ethiopia	79	6.6%	52.9 (8.5)	28 (4.5)	38.6 (7.1)	14	33.4 (6.1)	5 (35.7%)
Cameroon	70	5.9%	42 (8.5)	30.4 (4.7)	37.5 (5.2)	52	30.7 (4.6)	30 (57.7%)
Zambia	58	4.9%	45.6 (12.8)	26.2 (3)	36.2 (9.6)	26	27.2 (3.5)	10 (38.5%)
South Africa	38	3.2%	52.7 (8.5)	26.8 (3)	37.4 (7.8)	1	27	0
Sudan	31	2.6%	51.5 (11.1)	26.6 (4.6)	39.3 (7.5)	6	31.5 (8)	1 (16.7%)
Liberia	22	1.8%	49.6 (12.5)	27.6 (3.4)	39.6 (10.2)	7	26.1 (2.8)	3 (42.9%)
Other (*n* = 17)^a^	102	8.5%	49.4 (13.3)	28.2 (4.3)	36.7 (6.7)	36	30.9 (5.2)	15 (41.7%)
Total	1,194	100%	53.1 (11.1)	27.1 (3.9)	37.3 (8.7)	224	30 (5.2)	109 (48.7%)

Data source: American Medical Association [115].

a Each of these 17 sub-Saharan African countries had ≤15 IMGs trained outside sub-Saharan Africa in the 2011 AMA Physician Masterfile.

The mean graduation age (29.9 y, SD = 5.1) among IMGs in the 2000–2010 cohort is comparable to that of USMGs (29.3 y, SD = 3.9), and suggests that many of these IMGs may have completed a 4-y undergraduate program of studies, possibly in the US, before entering medical school overseas. The distribution of primary specialty choices and residency matriculations among these IMGs is consistent with SSA-IMGs and SSA-USMGs (Figures S8, S9, S10). The three most popular specialty choices in this group are internal medicine (*n* = 255), family practice (*n* = 161), and pediatrics (*n* = 82), with the number of women pediatricians equaling that of men. Together, these three specialties represent 42.3% (*n* = 498) of all IMGs age ≤70 y. These IMGs have completed their residency training in over 400 US residency programs with Howard University Hospital training the highest number (*n* = 22).

## Discussion

We have characterized three groups of SSA émigré physicians in the 2011 AMA-PM: (1) SSA-IMGs, i.e., physicians who graduated from medical schools located in SSA (68%, *n* = 7,130); (2) SSA-USMGs, i.e., physicians who were born in SSA but went to medical school in the US (20%, *n* = 2,053); and (3) physicians who were born in SSA but graduated from foreign medical schools outside the US and SSA (12%, *n* = 1,194). Together, these 10,377 SSA émigrés represent <1.2% of physicians in the AMA-PM (*n* = 924,139, excluding inactive, retired, and semi-retired physicians), and <5% of IMGs reported in the AMA-PM (*n* = 248,008) [10]. However, 10,377 SSA physicians in the US is a significant brain drain for SSA; the number exceeds the total number of physicians in Ethiopia, Ghana, Liberia, Tanzania, Uganda, Zambia, and Zimbabwe combined (estimated at 9,560 from 2005–2010) [5]. These seven countries have significant fractions of their physicians in the US and a combined population exceeding 210 million people, equivalent to 0.05 physician for 1,000 people compared to 2.5/1,000 in the US in 2009, a 50-fold ratio [55].

Another striking example is that of Liberia. If only half of the Liberian physicians identified in the 2011 AMA-PM were to return to Liberia, their presence would more than double the Liberian physician workforce (Table 10). Likewise, if half of Ghanaian-trained physicians identified in the 2011 AMA-PM and Ghanaian-born émigrés who graduated from US medical schools were to return to Ghana, their presence would increase Ghana's physician workforce by >30%. Such hypotheticals are instructive to illustrate the magnitude of the brain drain burden, though we do not suggest that such a reverse migration would occur in the absence of improved facilities, drugs, supplies, support staff, financial conditions, and, perhaps, political reform. In some cases, IMGs returning home may face regulatory obstacles to have their US credentials recognized and obtain a license to practice [56].

**Table 10:** Aggregate stock and overall emigration fraction of Sub-Saharan African physicians in the US physician workforce in 2011.

Source Countries (Top 12)	SSA-IMGs^a^	SSA-USMGs^a^	Other IMGs^a^	Aggregate Stock of SSA Physicians in the 2011 AMA-PM	Physicians Reported in Source Countries	Overall Emigration Fraction (%)	Potentially Active SSA Physicians in the AMA-PM (Age ≤70)	“Active” Emigration Fraction (%)	Potential Gain in Source Countries in Case of 50% Return Migration
Liberia	56	96	23	175	51	77.4%	170	76.9%	166.7%
Ghana	721	404	118	1,243	2,033	37.9%	1,226	37.6%	30.2%
Tanzania	24	35	95	154	300	33.9%	141	32.0%	23.5%
Ethiopia	531	240	91	862	2,152	28.6%	844	28.2%	19.6%
Zambia	81	82	59	222	836	25.5%	221	25.4%	17.0%
Zimbabwe	112	43	9	164	827	16.5%	155	15.8%	9.4%
Cameroon	63	66	70	199	1,346	12.9%	199	12.9%	7.4%
Kenya	173	296	394	863	7,549	10.3%	830	9.9%	5.5%
Uganda	145	70	126	341	3,361	9.2%	321	8.7%	4.8%
Nigeria	3,271	407	139	3,817	55,376	6.4%	3,763	6.4%	3.4%
South Africa	1,786	178	43	2,007	38,236	5.0%	1,789	4.5%	2.3%
Sudan	329	31	43	403	10,813	3.6%	390	3.5%	1.8%
Other (*n* = 16)	78	178	113	369	21,666	1.7%	328	1.5%	0.8%
Total	7,370	2,126	1,323	10,819	144,546	7.0%	10,377	6.7%	3.5%

Data sources: World Health Organization [5]; American Medical Association [115].

a SSA-IMGs, sub-Saharan African-trained medical graduates; SSA-USMGs, US-trained medical graduates born in sub-Saharan Africa; Other IMGs, sub-Saharan African-born internationals medical graduates trained outside sub-Saharan Africa.

A near doubling from the 5,336 SSA émigré physicians since 2002 [19] to 10,819 in 2011, using the same AMA source with its incomplete and missing data, suggests: (a) a probable underestimation of the actual number of SSA migrant physicians integrated into the US physician workforce; and (b) a serious continuing depletion of scarce human resources from SSA if current patterns were to continue. SSA has only 2% of the global physician workforce [57], yet bears 24% of the global burden of diseases including 69% of the world's HIV/AIDS infections [58]. The SSA region needs an estimated 420,000 additional physicians by 2015 in order to meet health-related targets for the millennium development goals [57]. As US medical schools awarded 29,775 medical degrees from 1978 to 2008 to Blacks/African Americans [59], we roughly estimate the population of SSA physicians age ≤70 identified in the 2011 AMA-PM to represent between 22.4% (*n* = 8,588 when excluding South African physicians) and 27.3% (*n* = 10,377 when including South African physicians) of the “black” physician workforce in the US. While we are unaware of any specific study of SSA physicians' clientele and outcomes in the US, studies of IMGs in general suggest they are more likely to practice in underserved communities than are USMGs [60]–[63]. Similarly, black physicians are more likely to practice in underserved communities than are whites [64]–[71], so one can speculate that SSA doctors are disproportionately valuable to a US health system plagued with geographic maldistributions. This may be a motivation for US institutions, notably historically black colleges and universities like Howard University and Meharry Medical College (Figure 9), or Harlem Hospital Center to recruit Africans for their medical schools and/or training programs.

Certification data reported by the ECFMG on IMGs seeking residency admission in the US suggest that only 57% of applicants succeeded in obtaining ECFMG certification between 1986 and 2005 [32]. Thus, there is a sizable but unknown number of SSA-trained physicians who may be in the US but are not practicing medicine, having not passed ECFMG certification (such SSA physicians would not be captured in the AMA-PM). Hence, even if the migration of SSA-trained physicians to the US may be interpreted as a loss from the perspective of the African countries, it cannot automatically be interpreted to be an addition to the US health care system. The failure in the destination country to practice within the profession for which they were trained implies “brain waste” for many migrant physicians according to Mattoo et al. [72]. They further find that skilled émigrés from developing countries where English is not the language of education suffer the most from “under-placement” or low transferability of skills following their entry into the US labor market. This implies that SSA physicians from non-English speaking countries (i.e., Angola, Benin, French-speaking Cameroon, DRC, Mali, Mozambique, Senegal, etc) may be at greater risk of failing ECFMG certification and experiencing brain waste upon immigrating to the US. However, this was beyond the scope of our investigation, as was any information about the skills of IMGs [73],[74], their issues of adjustment to US culture [75],[76], and the competing claims on the evolving shortage of the US physician workforce and the need for IMGs [77]–[79].

### Structural Adjustment Programs and the SSA Physician Brain Drain

Our study suggests that the physician brain drain from SSA to the US began in earnest in the mid-1980s and accelerated in the 1990s during the implementation years of the SAPs. Briefly, the SAPs were a set of conditionalities for refinancing (i.e., taking new loans to service outstanding debts) imposed by international financial institutions, principally the International Monetary Fund (IMF) and the World Bank, to debt-burdened developing countries at the outset of the Third World debt crisis in the early 1980s [52]–[54]. Grounded in the free market ethos of the Washington Consensus [80], these macro-economic conditionalities included among other things, local currency devaluation, desubsidization of public sector services (e.g., education, transportation, public works, health), privatization of state-owned industries, and deregulation or market liberalization to facilitate the free flow of commerce and promote more competition within the private sector [81]–[86]. In Africa, this translated into deep cuts to basic public sector health care services; imposition of fees for health care provision and education; near obliteration of health research budgets; extended freezes in public sector hiring, including public education and public health sectors; unprecedented pauperization of academic and public health sector staff; increases of social inequalities and economic vulnerability; and the mushrooming of international non-governmental organizations, often with minimal accountability to the local authorities [53],[54],[81]–[85].

The human cost and socio-economic consequences of the SAPs have been discussed extensively and widely criticized on both political and moral grounds [86]–[95]. In Zimbabwe, the reductions in public spending on health care in the 1990s triggered high health worker attrition and expatriation, resulting in a 30% drop in the quality of health care provision when compared to the achievements from the post-independence period [92]. In the seminal *Adjustment with a Human Face*, the UNICEF office in Ghana reported that Ghana “lost more than 50 per cent of its physicians between 1981 and April 1984, and about 8.5 per cent of nurses in 1982 alone” [94].

In sum, most SAPs failed because of poverty and the inability of patients to pay, with a consequent deterioration of the public sector health system due to poor financing and loss of personnel. While the implementation of the SAPs may have ended officially in the late 1999 with the adoption by international financial institutions of a new Poverty Reduction Strategy Paper approach [95], their effects, of which large-scale skilled migration is one, have been pervasive.

### Shortened Service and Heightened Emigration Trends among Recent Graduates

While migrant physicians who graduated in the 1980s and the 1990s may have left their native SSA countries in response to the low salaries and poor working conditions exacerbated by dysfunctional SAPs, emigration trends observed among SSA physicians who graduated in the new millennium reflect different dynamics. We found that earlier émigrés arrived in the US 8 y after graduation, on average, compared to 2.4 y for the later émigrés. The sharp decline in the number of years served prior to emigration reflects the increasingly fast pace of medical migration among newly graduated SSA-IMGs and foretells the challenges of stemming the medical brain drain in an era of increased globalization.

Using 1985–1994 University of Ghana graduation records, Dovlo and Nyonator [96] estimated that on average 50% of their medical graduates emigrated within 4.5 y and 75% within 9.5 y. In a 2009 study of the financial consequences of African medical migration, Clemens [97] surveyed a sample of 1,159 migrant physicians from Africa located in Canada and the US, and estimated that, on average, African-trained physicians immigrated to North America 7.2 y after receiving their medical degrees. However, the author conflated arrival data of North African IMGs with those of SSA-IMGs, thus making it difficult to determine SSA-IMGs' mean year of service prior to emigration. Nevertheless, Clemens' and Dovlo's studies further underscore the importance of the length of service by physicians in their home countries before emigration.

Our estimations suggest that, on average, SSA-IMGs graduate from medical school at age 25 and immigrate to the US at age 31.5, suggesting a potential length of service of 6.5 y prior to emigration. However, if current trends from West Africa and East Africa are sustained, it is expected that migrant physicians' mean year of graduation and mean year of emigration will continue to converge, portending an even grimmer health worker crisis in SSA in the near future. In fact, this trend of shortened service and heightened immigration is likely to be extended to other technical experts (e.g., engineers and scientists) if the arguments in the US for visa liberalization for highly educated persons are translated into law as fervently advocated in a new book [98] and in a recent testimony in front of the US House Committee Judiciary [99]. These arguments are epitomized by the US-centric views of a columnist for *The New York Times*: “Right now we have thousands of foreign students in America and one million engineers, scientists and other highly skilled workers here on H-1B temporary visas, which require them to return home when the visas expire. That's nuts” [100].

The view that the US should expand immigration to address its shortages of highly trained professionals and innovators is already codified in the current ease with which foreign physicians can secure visas and permanent residency if they pass ECFMG examinations and agree to provide care in a US physician-shortage area. Such initiatives as the General Agreement on Trade in Services (GATS), a global treaty within the World Trade Organization, have viewed the service sector much as the General Agreement on Tariffs and Trade (GATT) views merchandise trade, breaking down visa barriers (as with tariffs on goods) to smooth globalization and cross-border economic integration [101]–[103]. With continued globalization, we expect increased SSA physician emigration unless we can improve SSA physician numbers and job satisfaction substantially in their home countries. Such programs as the PEPFAR and NIH-supported Medical Education Partnership Initiative (MEPI) [14], and the smaller Consortium of New Southern African Medical Schools [104], are designed to do both and should be sustained and expanded.

However, in allocating funds to support medical education in SSA, close attention should be paid not only to medical schools with the institutional capability to manage scarce resources efficiently, but also with the commitment to serve the local community. To that end, it is critical to identify SSA-based medical schools that may have an institutional incentive to promote the emigration of their graduates. Hagopian et al. [105] observed a “culture” of medical migration in several Nigerian and Ghanaian university campuses. In these institutions, which included the University of Ibadan, a major recipient of MEPI funds and the top SSA doctor-exporting institution to the US (see Figure 3), medical faculty members did not only fail to discourage migration, they actively encouraged it, using their personal stories of emigration and return to exemplify the potential benefits of medical migration.

Given the implacability of human migration, and the fine line between immigration restrictions and potential violations of health workers' freedom of movement, “circular migration” has been recommended by the Institute of Medicine's Committee on the US Commitment to Global Health [106], and reiterated by the recent WHO Global Code of Practice on the International Recruitment of Health Personnel [3] as a means to facilitate the temporary return of émigré physicians to their homelands without losing re-entry privileges in the medical workforce of their host countries. A similar concept advocating the extended deployment of émigré medical staff to their home countries as well as specialists from the host countries via “health exchange programs” funded by the foreign entities benefiting from émigré physicians' services has been suggested [107]. However, the high institutional cost associated with such health exchange programs may be a major disincentive. This is equally the case for the Global Health Service Partnership, launched in 2013 as a diplomacy-influenced deployment of US health care professionals in Malawi, Tanzania, and Uganda [108],[109].

### Effect of September 11, 2001 on the US Immigration of SSA-IMGs

Our study provides some evidence of the resiliency of medical migration against restrictive US immigration policies in the aftermath of September 11, 2001. Among SSA countries with large stocks of migrant physicians in the US, only the number of South African physicians has decreased in the last decade, and there is no evidence that this decrease is associated with post-9/11 immigration restrictions. For several African countries such as Ghana, Ethiopia, Nigeria, and Sudan, physician emigration rate and migrant physician stocks in the US have increased significantly in the last decade. Indeed, the steep drop of medical graduation observed among SSA natives attending medical schools in the US during the first half of 2000–2010 is offset by a swift increase in medical graduations during the second half, and both trends appear consistent with post 9/11 enrollment decreases and increases reported among foreign students attending US universities [110]–[113].

### Strengths, Limitations, and Implications

Our study helps expand and update previous studies on this important and controversial issue [16],[19]–[22]. Our study sought to filter possible “outliers,” that is, potential retirees or inactive physicians, unlike these earlier studies. Our study further complements recent findings from Tankwanchi [34] who reports a critical physician age when migration is most likely among SSA-IMGs. If the number of SSA-IMGs immigrating to the US peaks between ages 28–31, that is, 3–6 y after SSA medical school graduation, and wanes at age 38, at which time >90% of SSA-IMGs have already settled in the US, then policies aimed at incentivizing SSA physicians to stay in their home countries should put a particular focus on early-career physicians age ≤38 y [34].

Our study has limitations. First, the >10,000 SSA born or trained physicians whom we identified in the 2011 AMA-PM do not represent the entire universe of SSA physicians who migrated to the US. It is reasonable to believe that a significant number of SSA physicians are present in the US, but they are employed in other occupations aside from medicine because of not qualifying for a US residency position. Thus, our emigration data are minimum estimates of the true magnitude of the physician “brain drain” from SSA to the US; if future research can include these “wasted brains” [72] in the calculation of the physician brain drain, the true burden on SSA can be better assessed. Second, we cannot fully account for physicians who are born in SSA but were trained outside Africa since only a fraction of this group actually reports their country of birth. In other words, the full size of SSA-born physicians in the US physician workforce is likely to be larger. Of course, this group includes many physicians who might have immigrated to the US as children with their parents, or came to the US to go to medical school. So, these numbers must be treated with more attention when included in the overall medical brain drain from Africa. Nevertheless, it is part of the calculus and the debate and requires further research.

Third, we cannot truly determine the mean length of stay in the US of SSA physicians as we do not have access to exit data on SSA physicians who return home. While the records from the AMA-PM are extensive enough to capture nearly all African physicians who formally practice medicine in the US, they do not inform us on the cross-section of foreign-born or foreign-trained doctors who permanently leave the US after their residency training or some years of practice in the US, but may still represent themselves in the AMA-PM. Hence, two methodological questions are worthy of future investigation: How can we accurately estimate the number of SSA émigré physicians present in the US, but not licensed to practice medicine? How can we collect exit data on SSA émigré physicians who ultimately leave the US in order to properly determine return migration rates and patterns among émigré physicians from specific SSA countries?

We believe that triangulation of physician data from diverse sources can begin to address questions that are outstanding. The ECFMG database contains demographic data on all foreign medical graduates who have attempted to establish their readiness for US graduate medical education through completion of the required ECFMG certification [48]. Despite multiple attempts, our efforts to access the ECFMG database in order to compare data on SSA physicians with those from the AMA-PM were unsuccessful. The WHO Global Code of Practice on the International Recruitment of Health Professionals [3] explicitly recommends the ongoing tracking of migrant health professional data. Thus, future investigations must overcome ECFMG reluctance to share data in order to triangulate AMA, ECFMG, and the US Census data to yield more comprehensive and accurate figures on SSA physicians in the US.

The emigration of physicians from human-capital constrained African countries to the US is the result of complexities in the labor markets for health care professionals in both the origin and destination countries. In the US and many other high income countries, rapidly aging populations and escalating medical expenses [114] are creating increased demand pressures in the labor markets of physicians that their own health systems seem unable to meet in the short term. This internal structural imbalance has resulted in the US providing generous professional incentives and favorable immigration policies to physicians (and nurses) from all over the world, luring them to emigrate. This process starts with the surfeit of primary care residency positions that are vacant due a dearth of USMGs, and continues with further career opportunities to practice in the US without visa troubles.

Despite the complexities of the international migration regimes, the movement of physicians from poor countries with limited training facilities to rich countries is a modern form of primary resource transfer and may be seen as the lower income nations subsidizing the education of higher nation physicians. International migration is increasingly likely in the face of globalized and integrated economies. Thus, policy proposals to address the inequities of global physician distribution will depend not only on credible data to influence the US and other doctor-importing high-income OECD countries to train larger number of physicians to meet their own workforce needs, but also on a social justice agenda and global health initiatives that promote the creation of opportunity structures necessary to enhance career development, improve workplace conditions, and encourage the recruitment, training, and retention of resources, talents, and skills in SSA and other LMIC source countries.

## Supporting Information

Alternative Language Abstract S1 **Abstract Translated into French by ABST.**
(DOC)

Alternative Language Abstract S2 **Abstract Translated into Arabic by Sherif O. Elhassan.**
(DOC)

Alternative Language Abstract S3 **Abstract Translated into Portuguese by Olivia C. Manders.**
(DOC)

Alternative Language Abstract S4 **Abstract Translated into Spanish by Elizabeth C. Prom-Wormley.**
(DOC)

Figure S1 **Age correlation between Sub-Saharan African-trained medical graduates with missing and complete birth country data.**
(XLS)

Figure S2 **Graduation age correlation between Sub-Saharan African-trained medical graduates with missing and complete birth country data.**
(XLS)

Figure S3 **Emigration age correlation between Sub-Saharan African-trained medical graduates with missing and complete birth country data.**
(XLS)

Figure S4 **Primary specialty correlation between Sub-Saharan African-trained medical graduates with missing and complete birth country data.**
(XLS)

Figure S5 **Linear relationship between the number of Sub-Saharan African-trained medical graduates and the number of Sub-Saharan African-born US medical graduates.**
(XLS)

Figure S6 **Linear relationship between the number of Sub-Saharan African-trained medical graduates and the number of Sub-Saharan African-born international medical graduates educated outside Sub-Saharan Africa.**
(XLS)

Figure S7 **Linear relationship between the number of Sub-Saharan African-born US medical graduates and the number of Sub-Saharan African-born international medical graduates educated outside Sub-Saharan Africa.**
(XLS)

Figure S8 **Linear relationship between the primary specialty choices of Sub-Saharan African-trained medical graduates and Sub-Saharan African-born US medical graduates.**
(XLS)

Figure S9 **Linear relationship between the primary specialty choices of Sub-Saharan African-trained international medical graduates and Sub-Saharan African-born international medical graduates educated outside Sub-Saharan Africa.**
(XLS)

Figure S10 **Linear relationship between the primary specialty choices of Sub-Saharan African-born US medical graduates and Sub-Saharan African-born international medical graduates educated outside Sub-Saharan Africa.**
(XLS)

Table S1 **Group statistics for comparing Sub-Saharan African-trained medical graduates with missing and complete birth country data.**
(DOC)

Table S2 **Independent samples test comparing Sub-Saharan African-trained medical graduates with missing and complete birth country data.**
(DOC)

Table S3 **Countries of birth and countries of education of Sub-Saharan-trained medical graduates with complete birth country data in the 2011 AMA Physician Masterfile.**
(DOC)

## Abbreviations

AMA-PM: American Medical Association Physician Masterfile
ECFMG: Educational Commission for Foreign Medical Graduates
IMG: international medical graduate
OECD: Organization of Economic Cooperation and Development
SAP: structural adjustment program
SD: standard deviation
SSA: sub-Saharan Africa
SSA-IMG: SSA medical graduate
USMG: US medical graduate
WHO: World Health Organization

## References

[1] World Health Organization (2003) The world health report 2003: shaping the future. Available: http://www.who.int/whr/2003/en/whr03_en.pdf. Accessed 14 February 2013.

[2] World Health Organization (2006) Working together for health: the world health report 2006. Available: http://whqlibdoc.who.int/publications/2006/9241563176_eng.pdf. Accessed 14 February 2013.

[3] World Health Organization (2010) The WHO global code of practice on the international recruitment of health personnel. Available: http://www.who.int/hrh/migration/code/code_en.pdf. Accessed 15 February 2013.

[4] Lee J-W (2006). Message from the Director-General. World Health Organization, editor. Working together for health: the world health report 2006. Geneva: World Health Organization. p. xiii.

[5] World Health Organization (2013) Global health workforce statistics. Available: http://www.who.int/hrh/statistics/hwfstats/en/ Accessed 30 July 2013.

[6] World Health Organization (1976) Health personnel and hospital establishments: Volume III. World Health Stat Annual 1973–1976. Geneva: World Health Organization.

[7] Mullan F, Frehywot S, Chen C, Greysen R, Wassermann T, et al (n.d.) The Sub-Saharan African medical school study: data, observation, and opportunity. Available: http://samss.org/samss.upload/documents/126.pdf. Accessed 14 February 2013.

[8] Foundation for Advancement of International Medical Education and Research – Region: ‘Africa.’ Available: https://imed.faimer.org/results.asp?country=&school=&currpage=1&cname=&city=&region=AF&rname=Africa&psize=25. Accessed 17 February 2013.

[9] University of Copenhagen and World Health Organization – AVICENNA Directories of medical schools. Available: http://avicenna.ku.dk/database/medicine. Accessed 14 May 2013.

[10] Redi-Med Data – Redi-med data interactive medical database system: American Medical Association and American Osteopathic Association Counts. Available: http://www.redimeddata.com/RediCounts.asp. Accessed 14 June 2013.

[11] United States Census Bureau – International Data Base. Available: http://www.census.gov/population/international/data/idb/informationGateway.php. Accessed 19 February 2013.

[12] Barclay A (2002) The political economy of brain drain at institutions of higher learning in conflict countries: Case of the University of Liberia. Afr Issues 30: 42–46.

[13] The Economist online (2011) Africa's impressive growth. Available: http://www.economist.com/blogs/dailychart/2011/01/daily_chart. Accessed 23 April 2013.

[14] Mullan F, Frehywot S, Omaswa F, Sewankambo N, Talib Z, et al (2012) The Medical Education Partnership Initiative: PEPFAR's effort to boost health worker education to strengthen health systems. Health Aff (Millwood) 31: 1561–72. 22778346 10.1377/hlthaff.2012.0219

[15] Mullan F, Frehywot S, Omaswa F, Buch E, Chen C, et al (2011) Medical schools in Sub-Saharan Africa. Lancet 377: 1113–1121. 21074256 10.1016/S0140-6736(10)61961-7

[16] Clemens MA, Pettersson G (2008) New data on African health professionals abroad. Hum Resour Health 6: 1 Available: http://www.human-resources-health.com/content/6/1/1. Accessed 14 February 2013. 18186916 10.1186/1478-4491-6-1 PMC2254438

[17] Clemens MA (2007) Do visas kill? Health effects of African health professional emigration (Working paper No 114). Washington (D.C.): Center for Global Development. Available: http://www.cgdev.org/files/13123_file_Clemens_Do_visas_kill_3_.pdf. Accessed 14 February 2013.

[18] Clemens MA (2011) The financial consequences of high-skill emigration: lessons from African doctors abroad. Plaza S, Ratha D, editors. Diaspora for development in Africa. Washington: The World Bank. pp. 165–182.

[19] Hagopian A, Thompson M, Fordyce M, Karin E, Johnson KE, et al (2004) The migration of physicians from sub-Saharan Africa to the United States of America: measures of the African brain drain. Hum Resour Health 2: 17 Available: http://www.human-resources-health.com/content/2/1/17. Accessed 14 February 2013. 15598344 10.1186/1478-4491-2-17 PMC544595

[20] Mullan F (2005) The metrics of the physician brain drain. N Engl J Med 353: 1810–1818. 16251537 10.1056/NEJMsa050004

[21] Docquier F, Barghava A (2007) A new panel dataset on physicians' emigration rates (1991–2004). Available: http://perso.uclouvain.be/frederic.docquier/filePDF/MBD1_Description.pdf. Accessed 14 February 2013.

[22] Dumont JC, Zurn P (2007) Immigrant health workers in OECD countries in the broader context of highly skilled migration. Int Migr Outlook doi:_10.1787/migr_outlook-2007-5-en

[23] Alkire S, Chen L (2005) Medical exceptionalism in international migration: should doctors and nurses be treated differently? Tamas K, Palme J, editors. Globalizing migration regimes: new challenges to transnational cooperation. Burlington: Ashgate Publishing Company. pp. 100–117.

[24] Castles S, Miller JM (2009) The age of migration: international population movements in the modern world. New York & London: Guilford Press.

[25] Buchan J, Sochalski J (2004) The migration of nurses: trends and policies. Bull World Health Organ 82 8: 587–594. 15375448 PMC2622934

[26] Dovlo D (2005) Taking more than a fair share? The migration of health professionals from poor to rich countries. PLoS Med 2: e109 doi:10.1371/journal.pmed.0020109 15916458 10.1371/journal.pmed.0020109 PMC1140940

[27] Bundred PE, Levitt C (2000) Medical migration: who are the real losers? Lancet 356: 245–246. 10963214 10.1016/S0140-6736(00)02492-2

[28] Eastwood JB, Conroy RE, Naicker S, West PA, Tutt RC, et al (2005) Loss of health professionals from sub-Saharan Africa: the pivotal role of the UK. Lancet 365: 1893–1900. 15924988 10.1016/S0140-6736(05)66623-8

[29] Murray CL, Vos T, Lozano R, Naghavi M, Flaxman AD, et al (2012) Disability-adjusted life years (DALYs) for 291 diseases and injuries in 21 regions, 1990–2010: a systematic analysis for the Global Burden of Disease Study 2010. Lancet 380: 2197–2223. 23245608 10.1016/S0140-6736(12)61689-4

[30] Omoleke SA (2013) Chronic non-communicable disease as a new epidemic in Africa: focus on the Gambia. Pan Afr Med J doi:10.11604/pamj.2013.14.87.1899. Available: http://www.panafrican-med-journal.com/content/14/87/full/. Accessed 1 May 2013. 10.11604/pamj.2013.14.87.1899 PMC3641923 23646223

[31] Holmes MD, Dalal S, Volmink J, Adebamowo CA, Njelekela M, et al (2010) Non-communicable diseases in Sub-Saharan Africa: the case for cohort studies. PLoS Med 7: e1000244 doi:10.1371/journal.pmed.1000244 20485489 10.1371/journal.pmed.1000244 PMC2867939

[32] Educational Commission on Foreign Medical Graduates - About ECFMG certification. Available: http://www.ecfmg.org/certification/index.html. Accessed 14 February 2013.

[33] Ratha D, Mohapatra S, Özden C, Plaza S, Shaw W, et al.. (2011) Leveraging migration Africa: remittances, skills, and investments. Washington (D.C.: The World Bank. 212 p.

[34] Tankwanchi SBA (2012) Doctors beyond borders: data trends and medical migration dynamics from Sub-Saharan Africa to the United States (doctoral dissertation). Nashville: Vanderbilt University.

[35] Mills AJ, Kanters S, Hagopian A, Bansbak N, Nachega J, et al (2011) The financial cost of doctors emigrating from Sub-Saharan Africa: human capital analysis. BMJ 343: d7031. 22117056 10.1136/bmj.d7031 PMC3223532

[36] American Medical Association - How the data elements of the AMA physician Masterfile are collected, maintained, and verified. Available: http://www.ama-assn.org/ama1/pub/upload/mm/eProfiles/mm/primarysource.pdf. Accessed 14 February 2013.

[37] McLafferty S (2012) Spatial error in geocoding physician location data from the AMA Physician Masterfile: implications for spatial accessibility analysis. Spat Spatiotemporal Epidemiol 3: 3–38. 10.1016/j.sste.2012.02.004 22469489

[38] Williams PT (1996) Quality of the family physician component of AMA Masterfile. J Am Board Fam Pract 9: 94–99. 8659271

[39] American Medical Association – More about AMA database licensing. Available: http://www.ama-assn.org/ama/pub/about-ama/physician-data-resources/ama-database-licensing/more-about-ama-database-licensing.page? Accessed 17 February 2013.

[40] Foundation for Advancement of International Medical Education and Research – Kigezi International School of Medicine. Available: https://imed.faimer.org/details.asp?country=905&school=&currpage=1&cname=UGANDA&city=&region=&rname=&mcode=905020&psize=25. Accessed 14 February 2013.

[41] Department of Higher Education, State of Maine – Non accredited colleges & universities list: ST – SY. Available: http://www.maine.gov/education/highered/Non-Accredited/st-sy.htm. Accessed 1 May 2013.

[42] Senegalaisement.com - Noms et prénoms Sénégalais. Available: http://www.senegalaisement.com/senegal/noms_et_prenoms.html. Accessed 4 June 2013.

[43] Chapman M (2005) Some medical degrees are ‘worthless’. BBC News November 5. Available: http://news.bbc.co.uk/2/hi/uk_news/4410020.stm. Accessed 5 June 2013.

[44] Curtis P (2005 November 7) GMC launches inquiries into private medical schools. The Guardian Available: http://www.guardian.co.uk/education/2005/nov/07/highereducation.uk2. Accessed 5 June 2013.

[45] St. Luke School of Medicine – SLSOM is open for learning. Available: http://www.stluke.edu/#Open. Accessed 1 May 2013.

[46] West J (2005) NTLA orders ‘bogus’ St. Luke University closed. Liberian Observer Available: http://quackfiles.blogspot.com/2005/05/ntla-orders-bogus-st-luke-university.html. Accessed 1 May 2013.

[47] allAfrica (2010) Liberia: bribes deal backfires on fake medical school. Available: http://allafrica.com/stories/201003240965.html. Accessed 14 February 2013.

[48] Boulet RJ, Norcini JJ, Whelan PG, Hallock AJ, Seeling SS (2006) The international medical graduate pipeline: recent trends in certification and residency training. Health Aff (Millwood) 25: 469–477 doi:10.1377/hlthaff.25.2.469 16522588 10.1377/hlthaff.25.2.469

[49] O'Brien RH (1958) The dawn of medical education in tropical Africa. J Med Educ 33: 553–563. 13564107

[50] O'Brien RH (1962) Medical education in tropical Africa progresses. J Negro Educ 33: 536–541.

[51] Lewinshon DE, Arnold PC (2010) Motives for migration of South African doctors to Australia since 1948. Med J Aust 192: 288–290. 20201765 10.5694/j.1326-5377.2010.tb03511.x

[52] Adepoju A (2004) Trends in international migration in and from Africa. Massey DS, Taylor JE editors. International migration: prospects and policies in a global market. New York: Oxford University Press. pp. 59–76.

[53] Cheru F (1992) Structural adjustment, primary resource trade, and sustainable development in sub-Saharan Africa. World Dev 20: 497–512.

[54] Loewenson R (1993) Structural adjustment and health policy in Africa. Int J Health Serv 23: 717–730. 8276531 10.2190/WBQL-B4JP-K1PP-J7Y3

[55] National Center for Health Statistics (2012) Health, United States, 2011: with special feature on socioeconomic status and health. Available: http://www.cdc.gov/nchs/data/hus/hus11.pdf. Accessed 14 February 2013. 22812021

[56] Elliott J (2012) India considers allowing expatriate doctors to practise back home. BMJ 344: e3235 doi:10.1136/bmj.e3235 22561068 10.1136/bmj.e3235

[57] Scheffler MR, Liu X J, Kinfu Y, Dal Poz RM (2008) Forecasting the global shortage of physicians: an economic-and needs-based approach. Bull World Health Organ 86: 516–523. 18670663 10.2471/BLT.07.046474 PMC2647492

[58] Joint United Nations Programme on HIV/AIDS (UNAIDS) Global report: UNAIDS report on the global AIDS epidemic 2012. Available: http://www.unaids.org/en/media/unaids/contentassets/documents/epidemiology/2012/gr2012/20121120_UNAIDS_Global_Report_2012_en.pdf. Accessed 14 February 2013.

[59] Association of American Medical Colleges (2010) Diversity in the physician workforce: facts & figures 2010. Available: https://members.aamc.org/eweb/upload/Diversity%20in%20the%20Physician%20Workforce%20Facts%20and%20Figures%202010.pdf. Accessed 14 February 2013.

[60] Howard DL, Bunch CD, Mundia WO, Konrad TR, Edwards LJ, et al (2006) Comparing United States versus international medical school graduate physicians who serve African- American and White elderly. Health Serv Res 41: 2155–2181. 17116114 10.1111/j.1475-6773.2006.00587.x PMC1955313

[61] Mick SS, Lee SY (1999) Are there need-based geographical differences between international medical graduates and U.S. medical graduates in rural U.S. counties? J Rural Health 15: 26–43. 10437329 10.1111/j.1748-0361.1999.tb00596.x

[62] Mick SS, Lee SY, Wodchis WP (2000) Variations in geographical distribution of foreign and domestically trained physicians in the United States: ‘safety nets’ or ‘surplus exacerbation’? Soc Sci Med 50: 185–202. 10619689 10.1016/s0277-9536(99)00183-5

[63] Thompson MJ, Hagopian A, Fordyce M, Hart LG (2009) Do international medical graduates (IMGs) “fill the gap” in rural primary care in the United States? A national study. J Rural Health 25: 124–134. 19785577 10.1111/j.1748-0361.2009.00208.x

[64] Brotherton SE, Stoddard JJ, Tang SS (2000) Minority and nonminority pediatricians' care of minority and poor children. Arch Pediatr Adolesc Med 154: 912–7. 10980795 10.1001/archpedi.154.9.912

[65] Cantor JC, Miles EL, Baker LC, Barker DC (1996) Physician service to the underserved: Implications for affirmative action in medical education. Inquiry 33: 167–80. 8675280

[66] Grumbach K, Hart LG, Mertz E, Coffman J, Palazzo L (2003) Who is caring for the underserved? A comparison of primary care physicians and nonphysician clinicians in California and Washington. Ann Fam Med 1: 97–104. 15040439 10.1370/afm.49 PMC1466573

[67] Komaromy M, Grumbach K, Drake M, Vranizan K, Lurie N, et al (1996) The role of Black and Hispanic physicians in providing health care for underserved populations. N Engl J Med 334: 1305–1310. 8609949 10.1056/NEJM199605163342006

[68] Moy E, Bartman BA (1995) Physician race and care of minority and medically indigent patients. JAMA 273: 1515–1520. 7739078

[69] Saha S, Komaromy M, Koepsell TD, Bindman AB (1999) Patient-physician racial concordance and the perceived quality and use of health care. Arch Intern Med 159: 997–1004. 10326942 10.1001/archinte.159.9.997

[70] Xu G, Fields SK, Laine C, Veloski JJ, Barzansky B, et al (1997) The relationship between the race/ethnicity of generalist physicians and their care for underserved populations. Am J Pub Health 87: 817–822. 9184512 10.2105/ajph.87.5.817 PMC1381056

[71] Walker KO, Moreno G, Grumbach K (2012) The association among specialty, race, ethnicity, and practice location among California physicians in diverse specialties. J Natl Med Assoc 104: 46–52. 22708247 10.1016/s0027-9684(15)30126-7 PMC3978451

[72] Mattoo A, Neagu CI, Özden C (2008) Brain waste? Educated immigrants in the US labor market. J Econ Dev 87: 255–269.

[73] Norcini JJ, Boulet JR, Dauphinee WD, Opalek A, Krantz ID, Anderson ST (2010) Evaluating the quality of care provided by graduates of international medical schools. Health Aff (Millwood) 29: 1461–1468. 20679648 10.1377/hlthaff.2009.0222

[74] Blonski J, Rahm S (2003) The relationship of residency performance to match status and US versus international graduate status. Fam Med 35: 100–104. 12607806

[75] Meghani SH, Rajput V (2011) Perspective: the need for practice socialization of international medical graduates–an exemplar from pain medicine. Acad Med 86: 571–574. 21436666 10.1097/ACM.0b013e318212e08b

[76] Morris AL, Phillips RL, Fryer GE, Green LA, Mullan F (2006) International medical graduates in family medicine in the United States of America: an exploration of professional characteristics and attitudes. Hum Res Health 4: 17 Available: http://www.human-resources-health.com/content/4/1/17. Accessed 14 February 2013. 10.1186/1478-4491-4-17 PMC1543651 16848909

[77] Goodman DC, Grumbach K (2008) Does having more physicians lead to better health system performance? JAMA 299: 335–337. 18212319 10.1001/jama.299.3.335

[78] Cooper RA (2004) Weighing the evidence for expanding physician supply. Ann Intern Med 141: 705–714. 15520427 10.7326/0003-4819-141-9-200411020-00012

[79] Salsberg E, Rockey PH, River KL, Brotherton SE, Jackson RG (2008) US residency training before and after the 1997 Balanced Budget Act. JAMA 300: 1174–1180. 18780846 10.1001/jama.300.10.1174

[80] Williamson J (1993) Democracy and the Washington consensus. World Dev 21: 1329–1336.

[81] Pfeiffer J, Chapman R (2010) Anthropological perspectives on structural adjustment and public health. Annu Rev Anthropol 39: 149–165.

[82] Moyo D (2009) Dead aid: why aid is not working and why there is a better way for Africa. New York: Farrar, Straus, & Giroux. 188 p.

[83] Maclean SJ, Quadir F, Shaw TM (1997) Structural adjustment and the response of civil society in Bangladesh and Zimbabwe: A comparative Analysis. N Polit Econ 2: 149–164.

[84] Moghadam V (2007) Gender and the global economy. Roberts JT, Hite AB, editors. The globalization and development reader: perspectives on development and global change. Malden: Blackwell Publishing. pp. 135––151.

[85] Brand H (1994) The World Bank, the Monetary Fund, and poverty. Int J Health Serv 24: 567–578. 7928019 10.2190/XHTR-48Q9-J3MU-1DGQ

[86] Lewis S (2006). Race against time. Toronto: Anansi. 216 p.

[87] Kolko G (1999) Ravaging the poor: The International Monetary Fund indicted by its own data. Int J Health Serv 29: 51–57. 10079397 10.2190/JG57-QQUD-MJ3G-291T

[88] Logie DE, Woodroffe J (1993) Structural adjustment: the wrong prescription for Africa? BMJ 307: 41 Available: http://www.bmj.com/content/307/6895/41. Accessed 14 February 2013. 8343672 10.1136/bmj.307.6895.41 PMC1678484

[89] Lugalla JL (1995) The impact of structural adjustment policies on women's and children's health in Tanzania. Rev Afr Polit Econ 22: 43–53. 12290679 10.1080/03056249508704099

[90] Mullan F (2007) Health, equity, and political economy: A conversation with Paul Farmer. Health Aff (Millwood) 26: 1062–1068.

[91] Cheru F (2002) Debt, adjustment and the politics of effective response to HIV/AIDS in Africa. Third World Q 23: 299–312.

[92] The Structural Adjustment Participatory Review International Network (2004) Structural adjustment: The SAPRI report. The policy roots of economic crisis, poverty and inequality. London & New York: Zed Books. 242 p.

[93] Cornia AG, Jolly R, Stewart F (1987) Adjustment with a human face: protecting the vulnerable and promoting growth. Oxford: Clarendon Press. 344 p.

[94] Cornia AG, Jolly R, Stewart F (1987) Adjustment with a human face: country case studies. Oxford: Clarendon Press. 328 p.

[95] International Monetary Fund, International Development Association (2002) Review of the poverty reduction strategy paper (PRSP) approach: early experience with interim PRSPs and full PRSPs. Washington (D.C.): IMF/World Bank. 102 p.

[96] Dovlo D, Nyonator F (2001) Migration by graduates of the University of Ghana medical school: A preliminary rapid appraisal. Available: http://www.who.int/hrh/en/HRDJ_3_1_03.pdf. Accessed 14 February 2013. Accessed 16 February 2013.

[97] Clemens MA (2009) The financial consequences of high-skill emigration: New data on African doctors abroad. Available: http://siteresources.worldbank.org/INTPROSPECTS/Resources/334934-1110315015165/Clemens.pdf. Accessed 14 February 2013.

[98] Wadhwa V (2012) The Immigrant exodus: why America is losing the global race to capture entrepreneurial talent. Philadelphia: Wharton Digital Press. 101 p.

[99] C-Span Video Library – Immigration Policy, February 5, 2013: Witnesses testify on immigration reform proposals on what needs to be included in a comprehensive immigration bill. Available: http://www.c-spanvideo.org/event/214127. Accessed 18 February 2013.

[100] Friedman TL (2010) Start-ups, not bailouts. The New York Times Available: http://www.nytimes.com/2010/04/04/opinion/04friedman.html. Accessed 4 February 4 2013.

[101] Adlung R, Carzaniga A (2001) Health services under the General Agreement on Trade in Services. Bull World Health Organ 79 4: 352–364. 11357215 PMC2566402

[102] Aginam O (2007) “Predatory globalization?”: the World Trade Organization, General Agreement on Trade in Services, and migration of African health professionals to the West. Falola T, Afolabi N, editors. The human cost of African migration. New York: Routledge. pp. 65–77.

[103] Schaffer RE, Waitzkin H, Brenner J, Jasso-Aguilar R (2005) Global trade and public health. Am J Public Health 95: 23–34. 15623854 10.2105/AJPH.2004.038091 PMC1449846

[104] Eichbaum Q, Nyarango P, Bowa K, Odonkor P, Ferrão J, et al (2012) “Global networks, alliances and consortia” in global health education-the case for south-to-south partnerships. J Acquir Immune Defic Syndr 61: 263–4. 22878420 10.1097/QAI.0b013e31826bf957

[105] Hagopian A, Ofosu A, Fatusi A, Biritwum R, Essel A, et al (2005) The flight of physicians from West Africa: views of African physicians and implications for policy. Soc Sci Med 61: 1750–1760. 15927335 10.1016/j.socscimed.2005.03.027

[106] Committee on the US Commitment to Global Health, Board on Global Health (2009) The US commitment to global health: recommendations for the public and private sectors. Washington (D.C.): The National Academies Press. 298 p. 20662131

[107] Mackey TK, Liang BA (2012) Rebalancing brain drain: Exploring resource reallocation to address health worker migration and promote global health. Health Policy 107: 66–73. 22572198 10.1016/j.healthpol.2012.04.006

[108] SEED Global Health – The joint program. Available: http://seedglobalhealth.org/joint-program/. Accessed 19 May 2013.

[109] Kerry VB, Auld S, Farmer P (2010) An international service corps for health—an unconventional prescription for diplomacy. N Engl J Med 363: 1199–1201. 20860500 10.1056/NEJMp1006501

[110] Goodall H (2008) More foreign science and engineering grad students flock to U.S., new survey finds. Chron High Educ Available: http://chronicle.com/article/More-Foreign-Science-and/449/. Accessed 14 February 2013.

[111] Jacobson J (2003) Foreign-student enrollment stagnates. Chron High Educ Available: http://chronicle.com/article/Foreign-Student-Enrollment/19236/. Accessed 14 February 2013.

[112] McCormack E (2005) Enrollment of foreign students fall for a 2nd year. Chron High Educ A1–A45 Available: http://chronicle.com/article/Enrollment-of-Foreign-Students/30801/. Accessed 14 February 2013.

[113] McMurtrie B (2008) Foreign students pour back into the U.S. Chron High Educ 55: A1–A25 Available: http://chronicle.com/article/Foreign-Students-Pour-Back/15377/. Accessed 14 February 2013.

[114] Brill S (2013 March 4) Bitter pill: why medical bills are killing us. Time Magazine Available: http://www.time.com/time/magazine/article/0,9171,2136864,00.html. Accessed 7 June 2013.

[115] American Medical Association (2011) AMA Physician Masterfile. Chicago: American Medical Association.

