# Correction: Physician Emigration from Sub-Saharan Africa to the United States: Analysis of the 2011 AMA Physician Masterfile

**DOI:** 10.1371/annotation/64ffd514-00bb-4a5e-9e2e-584763637d14

**Published:** 2013-12-16

**Authors:** Akhenaten Benjamin Siankam Tankwanchi, Çağlar Özden, Sten H. Vermund

In Table 1, the numbers of US medical schools should have been 130 (circa 1970), 173 (circa 2010), and 43 (change), instead of 126, 174, and 21 respectively. Please see the corrected Table 1 here: 

**Figure:**
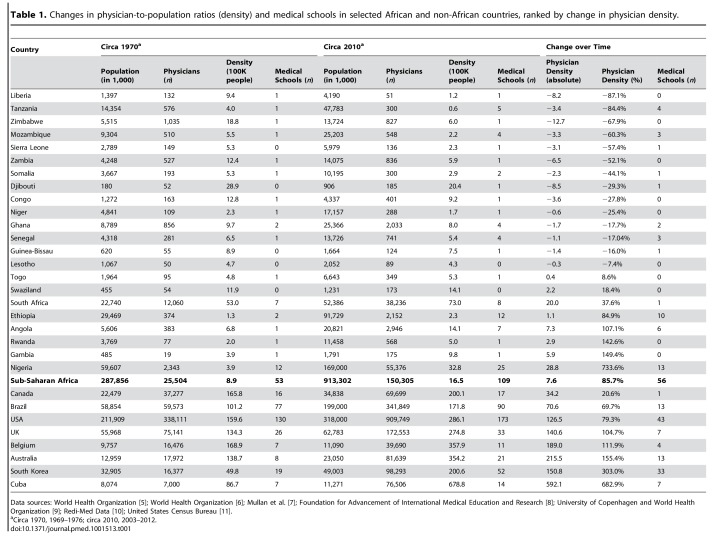


. 

