# Supplementary material for: Physician Emigration from Sub-Saharan Africa to the United States: Analysis of the 2011 AMA Physician Masterfile
Source: PLoS Med. 2013 Sep 17;10(9):e1001513. doi: 10.1371/journal.pmed.1001513 (PMC3775724; doi:10.1371/journal.pmed.1001513)
Supplement: Alternative Language Abstract S1 — Abstract Translated into French by ABST. (DOC) [file pmed.1001513.s001.doc]

**L’Émigration des Médecins de l’Afrique Subsaharienne vers les États-Unis: Une Analyse du Fichier des Médecins de l’Association Médicale Américaine de l’an 2011**

**Résumé**

**Contexte**: L’émigration de grande envergure des personnels de santé en provenance des pays en développement vers les pays à revenu élevé constitue un énorme frein au développement à court et à long terme desdits pays d’origine. Notre objectif est d’examiner de façon systématique les tendances migratoires récentes parmi les médecins originaires d’Afrique subsaharienne au sein du corps médical des États-Unis d’Amérique.

**Méthodes et Résultats**: Nous avons examiné les données des personnels de santé contenues dans l’Observatoire mondial de la Santé, conjointement aux statistiques collectées par l’Association Médicale Américaine (AMA) en 2011 et relatives à la présence dans le corps médical américain de médecins formés ou nés en Afrique subsaharienne. A partir des chiffres recueillis dans ces deux registres, nous avons estimé respectivement: les taux d’émigration; l’année d’immigration aux États-Unis; le nombre d’années d’ancienneté dans le pays d’origine avant l’émigration, et; la moyenne d’années passées aux États-Unis par les médecins issus des écoles de médecine d’Afrique subsaharienne. En 2011, nous avons identifié 10819 médecins formés ou nés dans 28 pays d’Afrique subsaharienne et recrutés dans le corps médical des États-Unis. Soixante-huit pour cent (n=7370) de ces immigrants ont été formés en Afrique subsaharienne; 20% (n=2126) ont été formés aux États-Unis, et; 12% (n=1323) ont été formés dans des pays étrangers autres que les États-Unis. Les médecins émigrés potentiellement actifs (âgés de ≤70 ans) représentent 96% (n=10377) du total. À l’exception de l’Afrique du Sud dont le nombre de médecins émigrés aux États-Unis a subit une baisse de 8% (-156) entre 2002 et 2011, l’émigration des médecins formés en Afrique subsaharienne recrutés dans le corps médical américain a augmenté de façon importante dans les autres principaux pays d’origine. Durant la dernière décennie, le nombre de médecins issus d’Afrique et intégrés dans le corps médical des États-Unis a accru de >50% pour ceux formés au Nigeria (+1113) et au Ghana (+243); de >100% (+274) pour ceux formés en Ethiopie, et; de >200 (+244) pour les médecins formés au Soudan. Avec un pourcentage d’émigration de 77% (n=175) en 2011, le Liberia est le pays Africain le plus affecté par l’émigration de ses médecins aux États-Unis. Les médecins formés en Afrique subsaharienne et identifiés dans le corps médical Américain en 2011 ont passé en moyenne 18 ans aux États-Unis. Après l’obtention de leur diplôme de médicine, ces derniers ont exercé en moyenne pendant 6.5 ans avant d’émigrer aux États-Unis. Près de la moitié ont émigré durant la période où leurs pays étaient soumis à des plans d’ajustement structurel (1984-1999).

**Conclusion**: À moins que des politiques ambitieuses relatives à l’exode des personnels de santé soient adoptées et mises en œuvre aux États-Unis et dans les pays d’origine desdits immigrants, les tendances migratoires actuelles perdureront. Les États-Unis resteront alors une destination prisée pour les médecins Africains émigrant du continent le plus en mal de ressources médicales et humaines.
