# Supplementary material for: Physician Emigration from Sub-Saharan Africa to the United States: Analysis of the 2011 AMA Physician Masterfile
Source: PLoS Med. 2013 Sep 17;10(9):e1001513. doi: 10.1371/journal.pmed.1001513 (PMC3775724; doi:10.1371/journal.pmed.1001513)
Supplement: Alternative Language Abstract S2 — Abstract Translated into Arabic by Sherif O. Elhassan. (DOC) [file pmed.1001513.s002.doc]

**هجرة الأطباء من دول جنوب الصحراء الكبرى الأفريقية إلى الولايات المتحدة: تحليل ملفات الأطباء الرئيسيه للجمعيه الطبيه الأمريكية لعام 2011**

**ملخص الدراسه:**

**المقدمه:**

إن هجرة الأطباء ذات النطاق الواسع من دول جنوب الصحراء الكبرى الأفريقية إلى الدول ذات الدخل المرتفع هو عامل مهم في التنميه والتطور. و هدف هذه الدراسه هو تقديم وصف شامل لتوجه هجرة الأطباء الحاليه من دول جنوب الصحراء الكبرى الأفريقية إلى الولايات المتحدة الأمريكية و التي وجد في القوى العاملة لأطباء.

**الأساليب والنتائج:**

قمنا بتحليل بيانات الأطباء من إحصائيات القوى العاملة للصحه العالمية بجانب بيانات التخرج و برنامج التخصص للأطباء الذين تدربوا أو ولدوا في دول جنوب الصحراء الكبرى الأفرقية و الذين يمارسون المهنة حالياً في الولايات المتحدة الأمريكية، وتم جمع هذه البيانات من ملفات الأطباء الرئيسية للجمعية الطبية الأمريكية لعام 2011. في هذه الدراسة تم تقدير نسبة المهاجرين، سنة دخول الولايات المتحدة الأمريكية، سنوات ممارسة المهنة قبل الهجرة، و مدة الإقامة في الولايات المتحدة. في ملف البيانات الرئيسية للجمعية الطبية الأمريكية لعام 2011، وجد أن 10,819 طبيب ولدوا أو تدربوا في 28 بلد أفريقي من دول جنوب الصحراء الكبرى الأفريقية. وكان هنالك 68% (ن = 7،370) تدربوا في دول أفريقيا ، و 20٪ تم تدريبهم في الولايات المتحدة الأمريكية (ن = 2،126)، و 12٪ (ن = 1،323) تدربوا خارج الولايات المتحدة الأمريكيه و دول جنوب الصحراء الكبرى الأفريقية. تم تقدير الأطباء النشطين (سن ≤ 70 عاما)، لتمثيل 96٪ (ن = 10،377) من المجموع. وجد في هذه الدراسة أن ميول الهجرة بين الأطباء المدربين في دول جنوب الصحراء الكبرى الأفريقية زاد من عام 2002 إلى عام 2011 في جميع ولكن لمصدر أساسي للبلد، وكان الاستثناء الوحيد لجنوب أفريقيا فعدد الأطباء المهاجرين إلى الولايات المتحدة انخفض بنسبة 8٪ (-156). وكانت زيادة عدد الأطباء المهاجرين في العشرة سنوات الأخيرة > 50٪ في نيجيريا (+1113) وغانا (+243)، >100٪ في إثيوبيا (+274)، و>200٪ (+244) في السودان. ليبيريا هي أكثر الدول التي تعاني من هجرة الأطباء إلى الولايات المتحدة الأمريكيه بنسبة 77٪ (ن = 175) من الأطباء المقدرة في الملفات الرئيسية للأطباء بالجمعية الطبية الأمريكية لعام 2011. من المعتاد لدى الأطباء المدربين في دول جنوب الصحراء الأفريقية أن يقيمون في الولايات المتحدة الأمريكيه لمدة 18 عاما. و أن أغلبهم كانوا يمارسون المهنه لمدة 6.5 سنوات قبل دخولهم الولايات المتحدة الأمريكيه، و قد هاجر ما يقارب من نصف الأطباء خلال سنوات تنفيذ برنامج التكيف الهيكلي (1984-1999).

**الخلاصه:**

ما لم يتم تنفيذ سياسات بعيدة المدى من قبل الولايات المتحدة الأمريكيه ودول جنوب الصحراء الكبرى الأفريقية، فإن رغبة الهجرة الحالية لا تزال قائمة، وسوف تظل الولايات المتحدة الأمريكية تشكل الوجهة الرائدة لأطباء دول جنوب الصحراء الأفريقية برغم أن يهاجرون من قارة هي بأشد الحاجة إليهم.
