# Supplementary material for: Physician Emigration from Sub-Saharan Africa to the United States: Analysis of the 2011 AMA Physician Masterfile
Source: PLoS Med. 2013 Sep 17;10(9):e1001513. doi: 10.1371/journal.pmed.1001513 (PMC3775724; doi:10.1371/journal.pmed.1001513)
Supplement: Alternative Language Abstract S3 — Abstract Translated into Portuguese by Olivia C. Manders. (DOC) [file pmed.1001513.s003.doc]

**A Emigração de Médicos da África Subsaariana para os Estados Unidos: Análise do Arquivo Mestre de Médicos de 2011**

**Abstrato**

**Fundo**: A emigração em grande escala de médicos da África Subsaariana (SSA) aos países de alta renda é um sério assunto de desenvolvimento. Nosso objetivo é fornecer uma descrição detalhada das tendências atuais de emigração de médicos dos SSA encontrados na força de trabalho médica dos Estados Unidos (EUA).

**Métodos e Resultados**: Analisamos dados nos médicos das Global Health Workforce Statistics (Estatísticas da Força de Trabalho em Saúde Global) juntamente aos dados de formatura e formação em residência do arquivo mestre de médicos de 2011 (American Medical Association Physician Masterfile [AMA-PM]) de médicos treinados ou nascidos em países da África Subsaariana que praticam correntemente nos EUA. Estimamos as proporções de emigração, ano de entrada aos EUA, anos de prática antes de emigração, e a duração de tempo nos EUA. No AMA-PM de 2011, encontramos 10.819 médicos nascidos ou formados de 28 países da África Subsaariana. Sessenta e oito por cento (n = 7.370) foram treinados na SSA, 20% (n = 2.126) treinados nos EUA, e 12% (n = 1.323) foram treinados fora tanto dos SSA como dos EUA. Estimamos que médicos ativos (idade ≤ 70 anos) representam 96% (n = 10.377) do total. As tendências migratórias entre os médicos treinados nos SSA aumentaram de 2002 e 2011 para todos os países de origem principal menos um; a exceção foi a África do Sul, cuja migração de médicos para os EUA diminuiu por 8% (-156). O aumento em última década migração foi mais de 50% na Nigéria (+1113) e no Gana (+243), mais de 100% na Etiópia (+274) e mais de 200% (+244) no Sudão. A Libéria é o mais aflito pela migração para os EUA com 77% (n = 175) de médicos estimados na 2011 AMA-PM. Na média, médicos formados nos SSA têm sido nos EUA por 18 anos. Eles praticaram por 6,5 anos antes da entrada aos EUA, e quase a metade emigraram durante os anos de implementação (1984-1999) dos programas de ajustamento estrutural.

**Conclusão**: A não ser que políticas abrangentes são implementadas pelos países norte-americanos e SSA, as tendências da emigração atuais persistirão, e os EUA continuarão a ser um destino primária para os médicos SSA que emigram do continente de maior necessidade.
