# Supplementary material for: Physician Emigration from Sub-Saharan Africa to the United States: Analysis of the 2011 AMA Physician Masterfile
Source: PLoS Med. 2013 Sep 17;10(9):e1001513. doi: 10.1371/journal.pmed.1001513 (PMC3775724; doi:10.1371/journal.pmed.1001513)
Supplement: Alternative Language Abstract S4 — Abstract Translated into Spanish by Elizabeth C. Prom-Wormley. (DOC) [file pmed.1001513.s004.doc]

**Emigración de médicos de África Subsahariana a Estados Unidos: Análisis del 2011 AMA Physician Masterfile**

**Abstracto**

**Antecedentes:** La gran escala de emigración de médicos con orígenes en África Subsahariana (SSA) a los países de altos ingresos es un problema developmental grave. Nuestro objetivo es mostrar una descripción detallada de las tendencias actuales de la emigración de los médicos de la SSA que son disponibles para trabajar en medicina dentro de los Estados Unidos (EEUU).
**Métodos y resultados:** Analizamos los datos de médicos usando las Estadísticas Globales de la Fuerza Laboral Para Ocupaciones de Salud con datos de graduación de escuela de medicina y de formación medica (residencia). Esta información viene del 2011 American Medical Association Masterfile (AMA-PM) de médicos formados o nacidos en países del África Subsahariana quienes estan practicando medicina en los EEUU. Estimamos proporciones de emigración, el año de entrada a los EEUU, el número de años de práctica antes de la emigración, y el tiempo de permanencia en los EEUU.

En AMA-PM del 2011, encontramos 10.819 médicos nacidos o formados en 28 países del África Subsahariana. Sesenta y ocho por ciento (n = 7.370) fueron formados en SSA, 20% (n = 2126) fueron educado en los EEUU, y el 12% (n = 1323) se formó fuera tanto SSA y los EEUU. Estimamos que los médicos activos (edad ≤ 70 años) representan el 96% (n = 10.377) del total. Las tendencias migratorias entre los médicos de SSA aumentó desde 2002 hasta 2011 en todos los países con la excepción de Sudáfrica cuya migración de médicos hacia los EEUU disminuyó un 8% (-156). El aumento de la migración en la última década fue > 50% en Nigeria (1113) y Ghana (243), > ​​100% en Etiopía (274), y > 200% (244) en Sudán. Liberia ha sido el más afectado por la migración a los EEUU con el 77% (n = 175) de sus médicos estimados en el 2011 AMA-PM. En promedio, los médicos formados en SSA han estado en los EEUU por 18 años. Practicaron por 6,5 años antes de entrar a los EEUU, y casi la mitad emigraron durante los años de aplicación (1984-1999) de los programas de ajuste estructural.
**Conclusión:** Si las políticas de largo alcance no están implementadas por los Estados Unidos y la SSA, las tendencias actuales de emigración persistirán, y los EEUU seguirá siendo un destino principal para los médicos de la SSA que emigran desde el continente de mayor necesidad.
