# Supplementary material for: Physician Emigration from Sub-Saharan Africa to the United States: Analysis of the 2011 AMA Physician Masterfile
Source: PLoS Med. 2013 Sep 17;10(9):e1001513. doi: 10.1371/journal.pmed.1001513 (PMC3775724; doi:10.1371/journal.pmed.1001513)
Supplement: Table S1 — Group statistics for comparing Sub-Saharan African-trained medical graduates with missing and complete birth country data. (DOC) [file pmed.1001513.s015.doc]

**Table S1. Group statistics for comparing Sub-Saharan African-trained medical graduates with missing and complete birth country data**

| **Selected variables** | **Birth country data** | **N** | **Mean** | **Std. deviation** | **Std. error mean** | |
| --- | --- | --- | --- | --- | --- | --- |
| **Age** | Missing | 5,171 | 48.86 | 10.838 | 0.151 |  |
|  | Complete | 2199 | 49.45 | 9.96 | 0.212 |  |
| **Age at graduation** | Missing | 5,171 | 24.99 | 2.322 | 0.032 |  |
|  | Complete | 2199 | 25.18 | 2.511 | 0.054 |  |
| **Year of graduation** | Missing | 5,171 | 1987 | 11.129 | 0.155 |  |
|  | Complete | 2,199 | 1987 | 9.926 | 0.212 |  |
