# Supplementary material for: Physician Emigration from Sub-Saharan Africa to the United States: Analysis of the 2011 AMA Physician Masterfile
Source: PLoS Med. 2013 Sep 17;10(9):e1001513. doi: 10.1371/journal.pmed.1001513 (PMC3775724; doi:10.1371/journal.pmed.1001513)
Supplement: Table S2 — Independent samples test comparing Sub-Saharan African-trained medical graduates with missing and complete birth country data. (DOC) [file pmed.1001513.s016.doc]

**Table S2. Independent samples test comparing Sub-Saharan African-trained medical graduates with missing and complete birth country data**

|  |  | **Levene's test for equality of variances** | | | **t-test for equality of means** | | | |  | **95% confidence** | | |
| --- | --- | --- | --- | --- | --- | --- | --- | --- | --- | --- | --- | --- |
|  |  | **F** | | **Sig.** | **t** | **df** | **Sig. (2-tailed)** | **Mean difference** | **Std. error difference** | **Lower** | **Upper** |  |
|  | Equal variances assumed | 31.081 | | .000 | -2.198 | 7372 | 0.028 | -0.592 | 0.269 | -1.12 | -0.064 |  |
| **Age** | Equal variances not assumed |  |  | | -2.274 | 4493.657 | 0.023 | -0.592 | 0.26 | -1.102 | -0.082 |  |
|  | Equal variances assumed | 13.682 | | .000 | -3.209 | 7370 | 0.001 | -0.194 | 0.061 | -0.313 | -0.076 |  |
| **Age at graduation** | Equal variances not assumed |  |  | | -3.109 | 3873.775 | 0.002 | -0.194 | 0.063 | -0.317 | -0.072 |  |
|  | Equal variances assumed | 52.259 | | .000 | 1.455 | 7370 | 0.146 | 0.399 | 0.274 | -0.139 | 0.937 |  |
| **Year of graduation** | Equal variances not assumed |  |  | | 1.524 | 4621.271 | 0.128 | 0.399 | 0.262 | -0.115 | 0.913 |  |
