# Supplementary material for: Physician Emigration from Sub-Saharan Africa to the United States: Analysis of the 2011 AMA Physician Masterfile
Source: PLoS Med. 2013 Sep 17;10(9):e1001513. doi: 10.1371/journal.pmed.1001513 (PMC3775724; doi:10.1371/journal.pmed.1001513)
Supplement: Table S3 — Countries of birth and countries of education of Sub-Saharan-trained medical graduates with complete birth country data in the 2011 AMA Physician Masterfile. (DOC) [file pmed.1001513.s017.doc]

**Table S3. Countries of birth and countries of education of Sub-Saharan-trained medical graduates (SSA-IMGs) with complete birth country data in the 2011 AMA Physician Masterfile**

|  | **Top 12 SSA countries of medical school** | | | | | | |  |  |  |  |  |  |  |  |
| --- | --- | --- | --- | --- | --- | --- | --- | --- | --- | --- | --- | --- | --- | --- | --- |
| **Birth countries (top 25)** | **Nigeria** | **Ghana** | **Ethiopia** | **SA** | **Sudan** | **Kenya** | **Uganda** | **Zimbabwe** | **Zambia** | **Cameroon** | **Liberia** | **Tanzania** | **Other** | **Total** |  |
| Nigeria | 553 |  |  |  |  |  | 1 |  | 1 |  |  |  | 1 | 556 |  |
| Ghana | 5 | 335 |  |  |  |  |  |  | 1 |  | 1 |  |  | 342 |  |
| Ethiopia |  |  | 251 |  | 1 | 1 | 1 |  |  |  |  |  | 1 | 255 |  |
| South Africa (SA) |  |  |  | 220 |  |  | 1 |  |  |  | 1 |  |  | 222 |  |
| Sudan |  |  |  |  | 124 |  |  |  |  |  |  |  |  | 124 |  |
| Kenya |  | 1 |  | 2 |  | 104 | 7 | 1 |  |  |  |  | 1 | 116 |  |
| US | 70 |  | 1 | 25 | 2 |  | 1 | 3 | 1 |  | 1 | 1 | 1 | 106 |  |
| UK | 33 | 2 |  | 18 |  |  |  | 2 | 4 |  |  |  |  | 59 |  |
| Zimbabwe |  |  |  | 36 |  |  |  | 21 | 1 |  |  |  |  | 58 |  |
| India | 28 | 1 |  | 2 |  | 1 | 5 |  | 18 |  |  | 1 |  | 56 |  |
| Uganda | 1 |  | 1 |  |  |  | 44 |  | 2 |  |  | 1 |  | 49 |  |
| Cameroon | 12 |  |  |  |  |  |  |  |  | 25 | 1 |  | 2 | 40 |  |
| South Korea |  |  |  | 31 |  |  | 1 |  |  |  |  |  |  | 32 |  |
| Zambia |  |  |  | 7 |  |  |  | 4 | 16 |  |  |  |  | 27 |  |
| Ukraine | 13 | 1 |  | 1 |  |  |  |  |  |  |  |  |  | 15 |  |
| Liberia |  |  |  |  |  |  |  |  |  |  | 14 |  |  | 14 |  |
| Tanzania | 1 |  |  |  |  | 1 | 4 |  |  |  |  | 6 |  | 12 |  |
| Somalia |  |  |  |  |  |  |  |  |  |  |  |  | 8 | 8 |  |
| Pakistan | 7 |  |  |  |  |  |  |  | 1 |  |  |  |  | 8 |  |
| Niger | 5 |  |  |  |  |  |  |  |  |  |  |  | 1 | 6 |  |
| Canada | 4 | 1 |  | 1 |  |  |  |  |  |  |  |  |  | 6 |  |
| Israel |  |  |  | 5 |  |  |  | 1 |  |  |  |  |  | 6 |  |
| Egypt | 1 |  | 1 |  | 2 |  |  |  | 1 |  |  |  |  | 5 |  |
| Germany | 1 |  |  | 3 |  |  |  |  |  |  |  | 1 |  | 5 |  |
| Senegal |  |  |  |  |  |  |  |  |  |  |  |  | 5 | 5 |  |
| Other (n=38) | 18 | 5 | 1 | 24 | 1 |  | 1 | 4 | 1 |  |  |  | 12 | 67 |  |
| Subtotal | 752 | 346 | 255 | 375 | 130 | 107 | 66 | 36 | 47 | 25 | 18 | 10 | 32 | 2,199 |  |
| Missing data | 2,519 | 375 | 276 | 1,411 | 199 | 66 | 79 | 76 | 34 | 38 | 38 | 14 | 46 | 5,171 |  |
| Total | 3,271 | 721 | 531 | 1,786 | 329 | 173 | 145 | 112 | 81 | 63 | 56 | 24 | 78 | 7,370 |  |
